# Supplementary material for: Mathematical modeling and parameter estimation of levodopa motor response in patients with parkinson disease
Source: PLoS One. 2020 Mar 3;15(3):e0229729. doi: 10.1371/journal.pone.0229729 (PMC7053720; doi:10.1371/journal.pone.0229729)
Supplement: S1 Material — (DOCX) [file pone.0229729.s001.docx]

**Supplementary Materials I: Model**

**Mathematical modeling and parameter estimation of levodopa motor response in patients with parkinson disease**

**Mauro Ursino^1*^, Elisa Magosso^1^**^¶^**, Giovanna Lopane^2,3^**^¶^**, Giovanna Calandra-Buonaura^2,3^**^&^**, Pietro Cortelli^2,3^**^&^**, Manuela Contin^2,3^**^¶^

*Individual neuron dynamics*

Let $i$ be a post-synaptic neuron, which receives synapses $w_{ij}$ from pre-synaptic neurons $j$, whose activity is $y_{j}$. Additional inputs coming from external sources are summarized in a single term $I_{i}$.

Every input to the neuron $i$ converges in a variable $x_{i}$. Assuming $N$ pre-synaptic neurons projecting to the post-synaptic neuron $i$, we can write:

$x_{i}=\sum_{j=1}^{N} w_{ij}y_{j}+I_{i}$ (1)

The input $x_{i}$ is then transformed into a post-synaptic variable $u_{i}$ using a first order differential equation with time constant *τ*, in order to mimic the cell membrane integrative process

$\tau\frac{du_{i}}{dt}=-u_{i}+x_{i}$ (2)

As last step, a sigmoidal function $\varsigma$ computes the activity of the neuron $i$, $y_{i}$, from the output of the previous differential equation $u_{i}$

$y_{i}=\varsigma(u_{i})$ (3)

implemented as

$y_{i}=\frac{1}{1+e^{-a(u_{i}-u_{0})}}$ (4)

being $a$ and $u_{0}$ parameters which set the central slope and the central position of the sigmoid.

*Network connectivity*

For the majority of the layers (S, C, Go, NoGo, Gpe, Gpi), synapses are represented with the symbol $W$ and two superscripts plus two subscripts. The two subscripts ($i$, $j$ with $i$, $j$ = 1, 2, …, $N$) specify the position of the post-synaptic and pre-synaptic neurons, respectively. $N$ denotes the number of action channels, i.e., the number of possible conflicting actions to be chosen. As in Baston *et al.* (2016) we use $N=2$, to represent the two possible actions of the alternate finger tapping test. Superscripts specify the target layer (where the post-synaptic neuron $i$ is located) and the donor layer (where the pre-synaptic neuron $j$ is located).

The STN and the cholinergic interneurons ChI are modeled as single units and therefore they do not need subscripts.

The acronyms are S: sensory cortex; C: motor cortex; T: thalamus; G: Go; N: NoGo; I: Gpi; E: Gpe; H: cholinergic interneurons ChI; STN: sub-thalamic nucleus. L indicates the lateral inhibition within the cortex.

The color of the arrows in Figure S1 represents the nature of the projections: excitatory green (or gray), inhibitory red (or black). Lateral inhibition is orange (light gray).

*Cortex -* We can write for $i=1,\ldots, N$:

$\tau_{L}\frac{du_{i}^{L}}{dt}=-u_{i}^{L}+\sum_{\begin{aligned} j=1 \\ i\neq j \end{aligned}}^{N} l_{ij}y_{j}^{C}$ (5)

$\tau\frac{du_{i}^{c}}{dt}=-u_{i}^{c}+\sum_{j=1}^{N} w_{ij}^{CS}s_{j}+u_{i}^{L}+w_{ii}^{CT}y_{i}^{T}+n_{i}$ (6)

$y_{i}^{C}=\varsigma(u_{i}^{c})$ (7)

where $y_{i}^{C}$ is the activity of a neuron of the cortex C. Every neuron of C receives excitatory inputs from the whole stimulus vector S (each individual component is named *s_j_*), an excitatory projection $y_{i}^{T}$ from the corresponding neuron in the thalamus and an additional input $u_{i}^{L}$, representing lateral inhibition from the other neurons in the cortex. A different time constant $\tau_{L}$ characterizes the latter. If the neuron of the thalamus is active, the neuron of the cortex receives the positive feedback and the corresponding action could be gated. *n_i_* is a Gaussian with noise with zero mean value and standard deviation, *σ_i_* , introduced during training to mimic exploration (which is an essential aspect of all learning processes).

*Go part of the striatum -* We can write for $i=1,\ldots, N$:

$\tau\frac{du_{i}^{G}}{dt}=-u_{i}^{G}+\sum_{j=1}^{N} w_{ij}^{GS}s_{j}+w_{ii}^{GC}y_{i}^{C}+\alpha\cdot D\cdot\left( y_{i}^{G}-\vartheta^{G} \right)+w^{GH}y^{H}$ (8)

$y_{i}^{G}=\varsigma(u_{i}^{G})$ (9)

where $y_{i}^{G}$ is the activity of a neuron of the Go part of the striatum. Every neuron of the Go receives excitatory input from the whole stimulus vector S and an excitatory projection from the corresponding neuron of the cortex C. The direct pathway starts here. Furthermore, activity of each neuron in the Go is modulated by dopamine (whose effect id denoted as *D*) and by the cholinergic interneuron activity ($y^{H}$).

Dopamine is excitatory ($\alpha>0$) if the Go activity is above threshold ($\vartheta^{G}$), inhibitory otherwise, thus realizing the contrast enhancement effect (Nicola *et al.*, 2000). The cholinergic interneurons are always inhibitory ($w^{GH}<0$) to the Go.

*NoGo part of the striatum -* We have for $i=1,\ldots, N$:

$\tau\frac{du_{i}^{N}}{dt}=-u_{i}^{N}+\sum_{j=1}^{N} w_{ij}^{NS}s_{j}+w_{ii}^{NC}y_{i}^{C}+\beta\cdot D+w^{NH}y^{H}$ (10)

$y_{i}^{N}=\varsigma(u_{i}^{N})$ (11)

where $y_{i}^{N}$ is the activity of a neuron of the NoGo part of the striatum. Every neuron of the NoGo receives excitatory input from the whole stimulus S and excitatory projection from the corresponding neuron in the cortex C. The indirect pathway starts here. Furthermore, activity of each neuron in the NoGo is modulated by dopamine and by the cholinergic interneuron activity ($y^{H}$).

Dopamine provides inhibition ($\beta<0$) to all the NoGo neurons, while the cholinergic interneuron provides excitation ($w^{NH}>0$). Hence, dopamine and acetylcholine exert specular tonic and phasic effects on NoGo activity with respect to the Go case.

*Globus pallidus pars externa -* Equations are for $i=1,\ldots, N$:

$\tau\frac{du_{i}^{E}}{dt}=-u_{i}^{E}+w_{ii}^{EN}y_{i}^{N}+w^{ESTN}y^{STN}+I^{E}$ (12)

$y_{i}^{E}=\varsigma(u_{i}^{E})$ (13)

where $y_{i}^{E}$ is the activity of a neuron of the Gpe. Every neuron of the Gpe receives an inhibitory projection from the corresponding neuron of the NoGo part of the striatum ($w_{ii}^{EN}<0$), and takes part to the indirect pathway. The excitation ($w^{ESTN}$) from the STN is part of a feedback loop to control STN activity. Due to the presence of an external input ($I^{E}$), every neuron of the Gpe is tonically active at rest.

*Globus pallidus pars interna* - Equations are for $i=1,\ldots, N$:

$\tau\frac{du_{i}^{I}}{dt}=-u_{i}^{I}+w_{ii}^{IG}y_{i}^{G}+w_{ii}^{IE}y_{i}^{E}+w^{ISTN}y^{STN}+I^{I}$ (14)

$y_{i}^{I}=\varsigma(u_{i}^{I})$ (15)

where $y_{i}^{I}$ is the activity of a neuron of the Gpi. Every neuron of the Gpi receives an inhibitory projection from the corresponding neuron of the Go part of the striatum ($w_{ii}^{IG}<0$), taking part to the direct pathway, and an inhibitory projection from the Gpe ($w_{ii}^{IE}<0$), while the excitation ($w^{ISTN}>0$) from the STN is part of the hyperdirect way. The STN excites all the neurons of the Gpi, which in turns inhibit the corresponding neurons in the thalamus, preventing action selection.

Every neuron of the Gpi is tonically active at rest. In fact, the external input ($I^{I}$) overcomes the inhibitory input coming from the Gpe: that is the reason why the Gpi is active in the tonic state and inhibits the thalamus, although the Gpe provides inhibition to the Gpi.

*Subthalamic nucleus -* Since $y^{STN}$ and $u^{STN}$ are scalar variables, we can write :

$\tau\frac{{du}^{STN}}{dt}=-u^{STN}+k^{E}E+\sum_{j=1}^{N} w_{J}^{STNE}y_{j}^{E}$ (16)

with

$E=\sum_{j=1}^{N} \sum_{\begin{aligned} i=1 \\ i\neq j \end{aligned}}^{N} y_{i}^{C}y_{j}^{C}$ (17)

$y^{STN}=\varsigma(u^{STN})$ (18)

where $y^{STN}$ is the activity of the STN. The STN is connected to the cortex C and computes the conflict within it by means of an energy function $E$. The latter rises when two or more neurons are simultaneously active in the cortex. This is how the hyperdirect pathway starts. The projection from the Gpe is part of the feedback loop that control STN activity.

*Thalamus -* We have for $i=1,\ldots, N$:

$\tau\frac{du_{i}^{T}}{dt}=-u_{i}^{T}+w_{ii}^{TI}y_{i}^{I}+w_{ii}^{TC}y_{i}^{C}$ (19)

$y_{i}^{T}=\varsigma(u_{i}^{T})$ (20)

where $y_{i}^{T}$ is the activity of a neuron of the thalamus. Every neuron of the thalamus receives an excitatory projection from the corresponding neuron of the cortex C ($w_{ii}^{TC}>0$), and an inhibitory projection from the corresponding neuron of the Gpi ($w_{ii}^{TI}<0$): the imbalance between the two projections establishes whether the corresponding action is gated or not. The excitation from the cortex to the thalamus realizes, together with the backward excitation from the thalamus to the cortex, a positive feedback loop, which is essential for cortical WTA mechanism.

Every thalamic neuron is tonically silent at rest, as a consequence of the tonic activity of the Gpi.

*Cholinergic interneurons -* Since $y^{H}$ and $u^{H}$ are scalar variables, we have:

$\tau\frac{{du}^{H}}{dt}=-u^{H}+I^{H}+\gamma\cdot D$ (21)

$y^{H}=\varsigma(u^{H})$ (22)

where $y^{H}$ is the activity of the cholinergic interneuron. The cholinergic interneuron is inhibited ($\gamma<0$) by dopamine ($D$) and is tonically active at rest thanks to the input $I^{H}$.

*Hebb rule*

Being $\Delta w_{ij}^{AB}$ the variation of the synapse between the pre-synaptic neuron $j$ in layer B (B = S or C) and the post-synaptic neuron $i$, in layer A (A = G or N), the Hebb rule is expressed as:

$\Delta w_{ij}^{AB}=\sigma{(y_{j}^{B}-\vartheta^{PRE})}^{+}(y_{i}^{A}-\vartheta^{POST})$ (23)

The first term compares pre-synaptic activities to specific thresholds (specified in Table S1).

The pre-synaptic term, thanks to the function “positive part” ([]+), detects where learning can occur: only the synapses coming from excited neurons of the cortex C or from salient stimuli in S, above the threshold $\vartheta^{PRE}$, can be modified. This means that only synapses from the chosen action (high value in C) and from the present context (high values in S) are subject to learning.

The post-synaptic term considers whether the post-synaptic activity $y_{i}$ (in our case Go or NoGo neuronal activity in the striatum) is above or below the threshold $\vartheta^{POST}$ (also specified in Table 1). Indeed, error feedbacks, i.e., rewards or punishments, cause a phasic change in the dopamine level (a peak or a dip, respectively), and subsequently a transient change in the ChI activity (Baston & Ursino, 2015a). As a consequence, striatal Go and NoGo neurons experience a phasic transient activity. The purpose of the threshold $\vartheta^{POST}$ is to detect when striatal phasic changes are occurring and evaluate the post-synaptic contribution at the correct time for proper synaptic learning.

The outcome of Hebbian learning is synaptic potentiation or depression.

As a fundamental biological constraint, we assume that synapses cannot change their sign (i.e., the nature of their neurotransmitter cannot be changed from excitatory to inhibitory or vice-versa), nor can increase above a maximum saturation value. Hence, we have, for each trained synapse:

$0\leq w_{ij}^{AB}\leq w_{max}$ (with A = G or N, and B = S or C). (24)

*Model of levodopa pharmaco-kinetics and pharmaco-dynamics*

The kinetics of levodopa was simulated as in the previous paper, where more details can found (Baston *et al.*, 2016).

A three-compartment model was adopted to describe plasma levodopa concentration. This corresponds to the following equations:

$V_{1}\frac{dc_{1}}{dt}=-\left( k_{21}+k_{31}+k_{e1} \right)c_{1}+k_{12}c_{2}+i$ (25)

$V_{2}\frac{{dc}_{2}}{dt}=k_{21}c_{1}-k_{12}c_{2}$ (26)

$V_{3}\frac{{dc}_{3}}{dt}=k_{31}c_{1}-k_{e3}c_{3}$ (27)

The first represents a central compartment, where levodopa is administered and plasma concentration is measured. The second is a peripheral compartment, representing the interaction between plasma and other body fluids. The third is an “effect compartment” simulating the concentration of the active metabolite on the basal ganglia. The model contains eight parameters: the inter-compartment rate constants ($k_{12}$ and $k_{21}$), the total body rate constant ($k_{etot}=k_{e1}+k_{31}$), the drug absorption from the central to the effect compartment and the drug removal from the effect compartment ($k_{31}$, and $k_{e3}$ respectively), and the compartment volumes ($V_{1}$ , $V_{2}$ and $V_{3}$).

Finally, we need a law describing how the concentration in the effect compartment (i.e., $c_{3}$) affects the dopaminergic term (named $D$ in Eqs. (8), (10) and (21)), representing how dopamine modulates the activity of the Go and NoGo neurons.

First, in order to account for the observed delay between plasma concentration and the clinical response, we introduced a pure delay, *T*. The delayed concentration is named $c_{3delay}$ . We have:

$c_{3delay}\left( t \right)= c_{3}\left( t-T \right)$ (28)

A classic way to describe the binding of a molecule with a receptor (or a reaction with cooperative effects, where an enzyme can bind one or more substrate molecules) is the Hill law (Keener & Sneyd, 2009). We can write:

$D=D_{0}+\frac{D_{max}c_{3delay}^{N_{D}}}{D_{c50}^{N_{D}}+c_{3delay}^{N_{D}}}$ (29)

where $D_{0}$ represents the basal value (i.e. the effect immediately before the beginning of levodopa administration) and the second term, with a sigmoidal shape, represents the effect induced by a levodopa concentration $c_{3}$, delayed by the time $T$. $D_{max}$ is the maximum effect that levodopa can produce, $D_{c50}$ is the concentration which produces 50% of the maximum effect, and $N_{D}$ is the Hill coefficient, which determines the slope of the concentration-effect relationship.

Figure S1


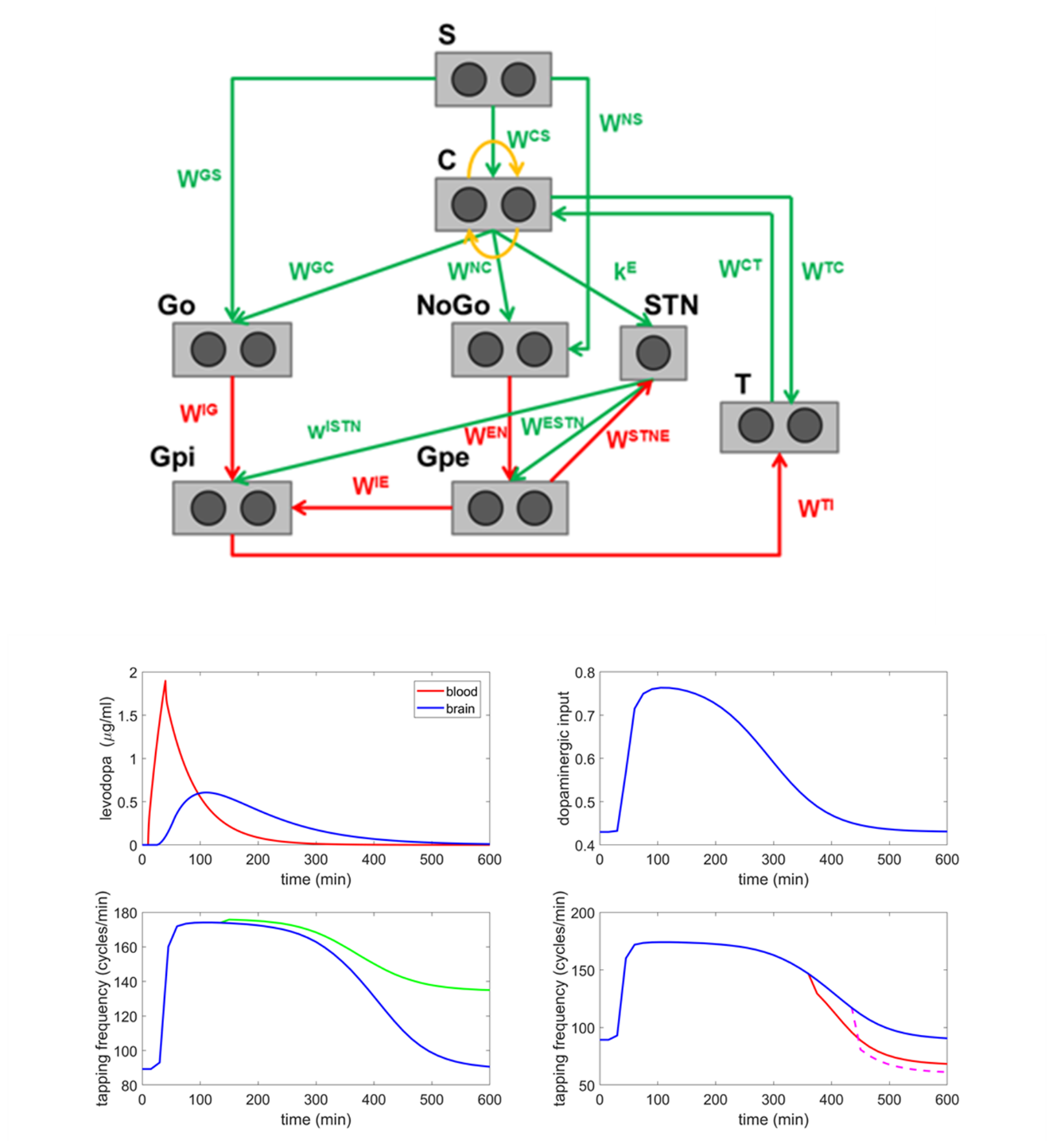


Table S1

Parameter values of the basal ganglia (BG) computational model related to Hebbian learning: $\sigma$ represents the gain, while $\vartheta^{PRE}$ and $\vartheta^{POST}$ are the thresholds to compute the pre-synaptic terms and the post-synaptic terms. The other parameters refer to the effect of dopamine and acethilcoline on striatal neurons, and to the neuron dynamics and sigmoid characteristics.

| Name | Value |
| --- | --- |
| $\boldsymbol{\tau}$/$\boldsymbol{\tau}_{\boldsymbol{L}}$ | 10[ms] / 5 [ms] |
| $\boldsymbol{a}$ | 4 |
| $\boldsymbol{u}_{\boldsymbol{0}}$ | 1 |
| $\boldsymbol{\vartheta}^{\boldsymbol{G}}$ | 0.35 |
| $\boldsymbol{I}^{\boldsymbol{E}}$ | 1 |
| $\boldsymbol{I}^{\boldsymbol{I}}$ | 3 |
| $\boldsymbol{I}^{\boldsymbol{H}}$ | 1.0 |
| $\boldsymbol{\alpha}$ | 0.75 |
| $\boldsymbol{\beta}$ | -1 |
| $\boldsymbol{\gamma}$ | -0.5 |
| $\boldsymbol{\sigma}$ | 0.01 |
| $\boldsymbol{\vartheta}^{\boldsymbol{PRE}}$ | 0.5 |
| $\boldsymbol{\vartheta}^{\boldsymbol{POST}}$ | 0.5 |
| $\boldsymbol{w}_{\boldsymbol{max}}$ | 1.2 |

Table S2

Synaptic values of the basal ganglia (BG) computational model before training, used to fit all patients. $W^{GC}$,$W^{GS}$, $W^{NC}$ and $W^{NS}$ are the synapses that are subject to training in Fig. 7. Before training, we used the parameter set derived in Ursino and Baston (2018) for a Parkinson-type subject. This is characterized by a moderate reinforcement of the Go, and moderate reduction of the NoGo.

| Name | Projection | Type | Values |
| --- | --- | --- | --- |
| $\boldsymbol{L}$ | inhibition | extradiagonal matrix | $l_{\begin{aligned} ij \\ i\neq j \end{aligned}}$= -1.2 |
| $\boldsymbol{W}^{\boldsymbol{CS}}$ | excitation | full matrix | $w_{ii}^{CS}$= 1.0; $w_{\begin{aligned} ij \\ i\neq j \end{aligned}}^{CS}$= 1.0 |
| $\boldsymbol{W}^{\boldsymbol{CT}}$ | excitation | diagonal matrix | $w_{ii}^{CT}$= 4 |
| $\boldsymbol{W}^{\boldsymbol{GC}}$ | excitation | diagonal matrix | $w_{ii}^{GC}$= 0.53 |
| $\boldsymbol{W}^{\boldsymbol{GS}}$ | excitation | full matrix | $w_{ii}^{GS}$= 0.53; $w_{\begin{aligned} ij \\ i\neq j \end{aligned}}^{GS}$= 0.4 |
| $\boldsymbol{W}^{\boldsymbol{NC}}$ | excitation | diagonal matrix | $w_{ii}^{NC}$= 0.46 |
| $\boldsymbol{W}^{\boldsymbol{NS}}$ | excitation | full matrix | $w_{ii}^{NS}$= 0.42; $w_{\begin{aligned} ij \\ i\neq j \end{aligned}}^{NS}$= 0.45 |
| $\boldsymbol{W}^{\boldsymbol{EN}}$ | inhibition | diagonal matrix | $w_{ii}^{EN}$= -2.2 |
| $\boldsymbol{W}^{\boldsymbol{IE}}$ | inhibition | diagonal matrix | $w_{ii}^{IE}$= -3 |
| $\boldsymbol{W}^{\boldsymbol{IG}}$ | inhibition | diagonal matrix | $w_{ii}^{IG}$= -36 |
| $\boldsymbol{W}^{\boldsymbol{TC}}$ | excitation | diagonal matrix | $w_{ii}^{TC}$= 3 |
| $\boldsymbol{W}^{\boldsymbol{TI}}$ | inhibition | diagonal matrix | $w_{ii}^{TI}$= -3 |
| $\boldsymbol{w}^{\boldsymbol{ESTN}}$ | excitation | scalar | $w^{ESTN}$ = 1 |
| $\boldsymbol{w}^{\boldsymbol{ISTN}}$ | excitation | scalar | $w^{ISTN}$ = 30 |
| $\boldsymbol{k}^{\boldsymbol{E}}$ | excitation | scalar | $k^{E}$ = 7 |
| $\boldsymbol{W}^{\boldsymbol{STNE}}$ | inhibition | row vector | $w_{i}^{STNE}$= -1 |
| $\boldsymbol{w}^{\boldsymbol{GH}}$ | inhibition | scalar | $w^{GH}$ = -1 |
| $\boldsymbol{w}^{\boldsymbol{NH}}$ | excitation | scalar | $w^{NH}$ = 1 |
