# Supplementary material for: Mathematical modeling and parameter estimation of levodopa motor response in patients with parkinson disease
Source: PLoS One. 2020 Mar 3;15(3):e0229729. doi: 10.1371/journal.pone.0229729 (PMC7053720; doi:10.1371/journal.pone.0229729)
Supplement: S2 Material — (DOCX) [file pone.0229729.s002.docx]

**Supplementary Materials II: Figures**

**Mathematical modeling and parameter estimation of levodopa motor response in patients with parkinson disease**

**Mauro Ursino^1*^, Elisa Magosso^1^**^¶^**, Giovanna Lopane^2,3^**^¶^**, Giovanna Calandra-Buonaura^2,3^**^&^**, Pietro Cortelli^2,3^**^&^**, Manuela Contin^2,3^**^¶^

**Group 1 (stable)**

**Patient 1**


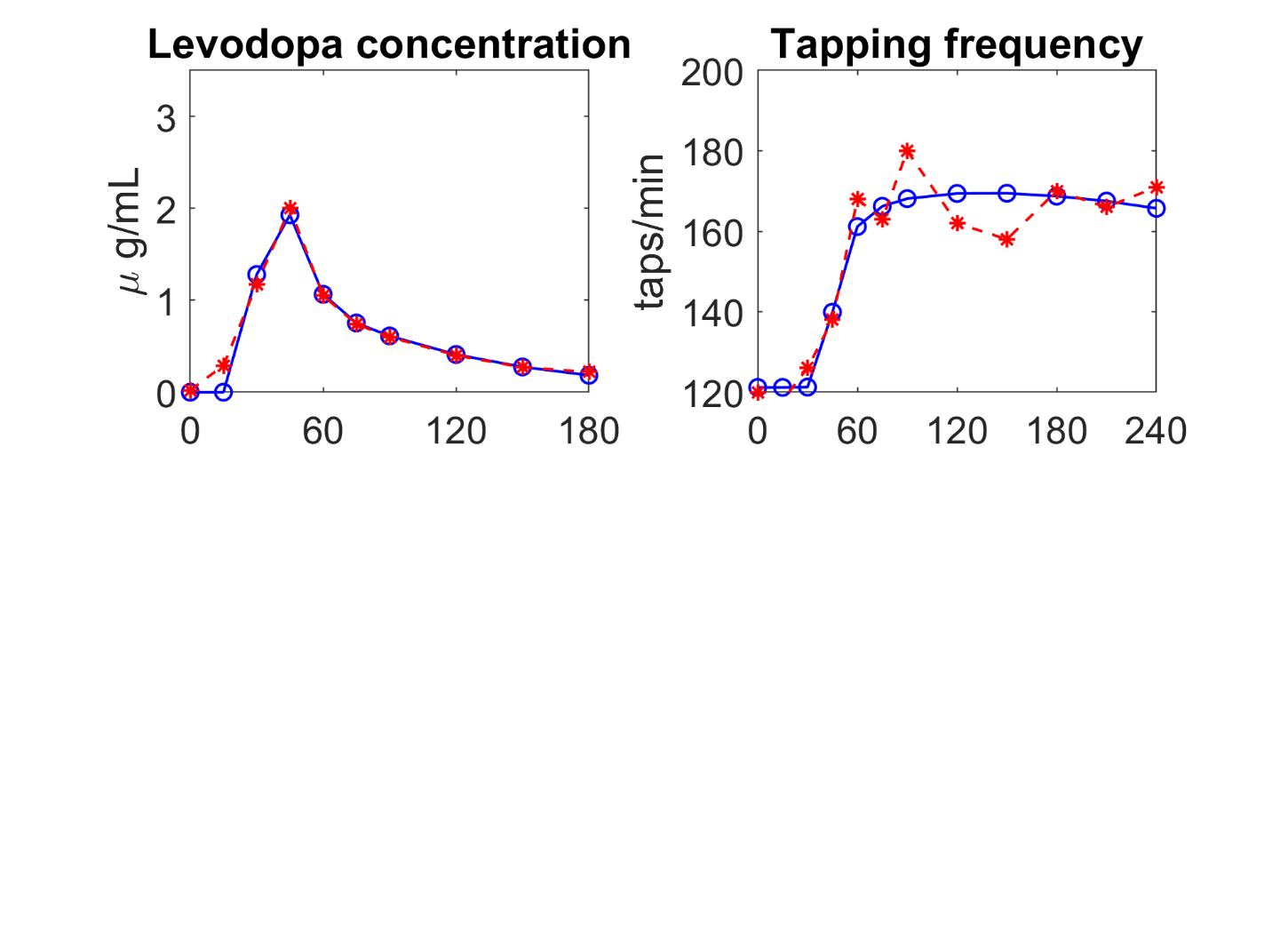


**Patient 2**


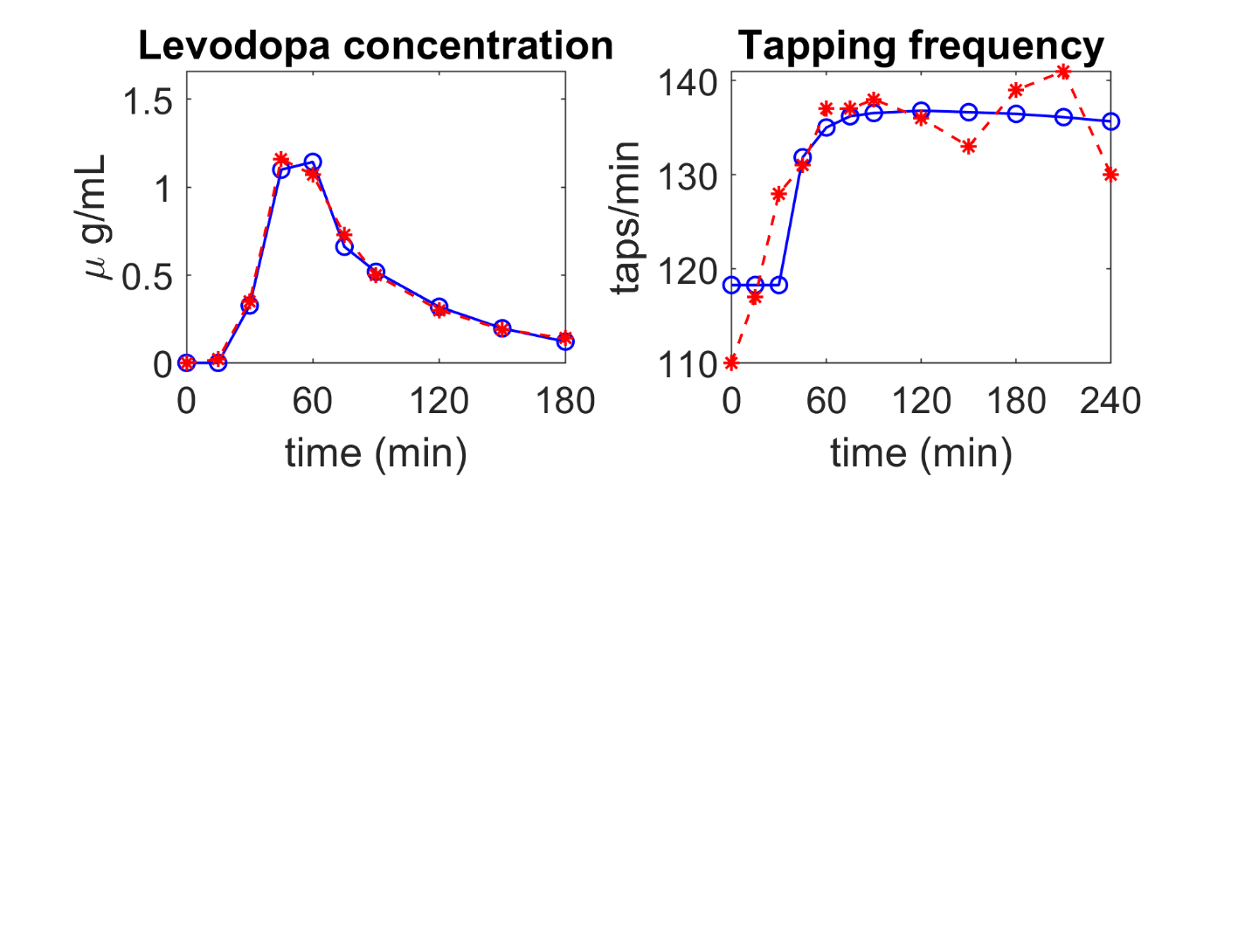


**Patient 3**


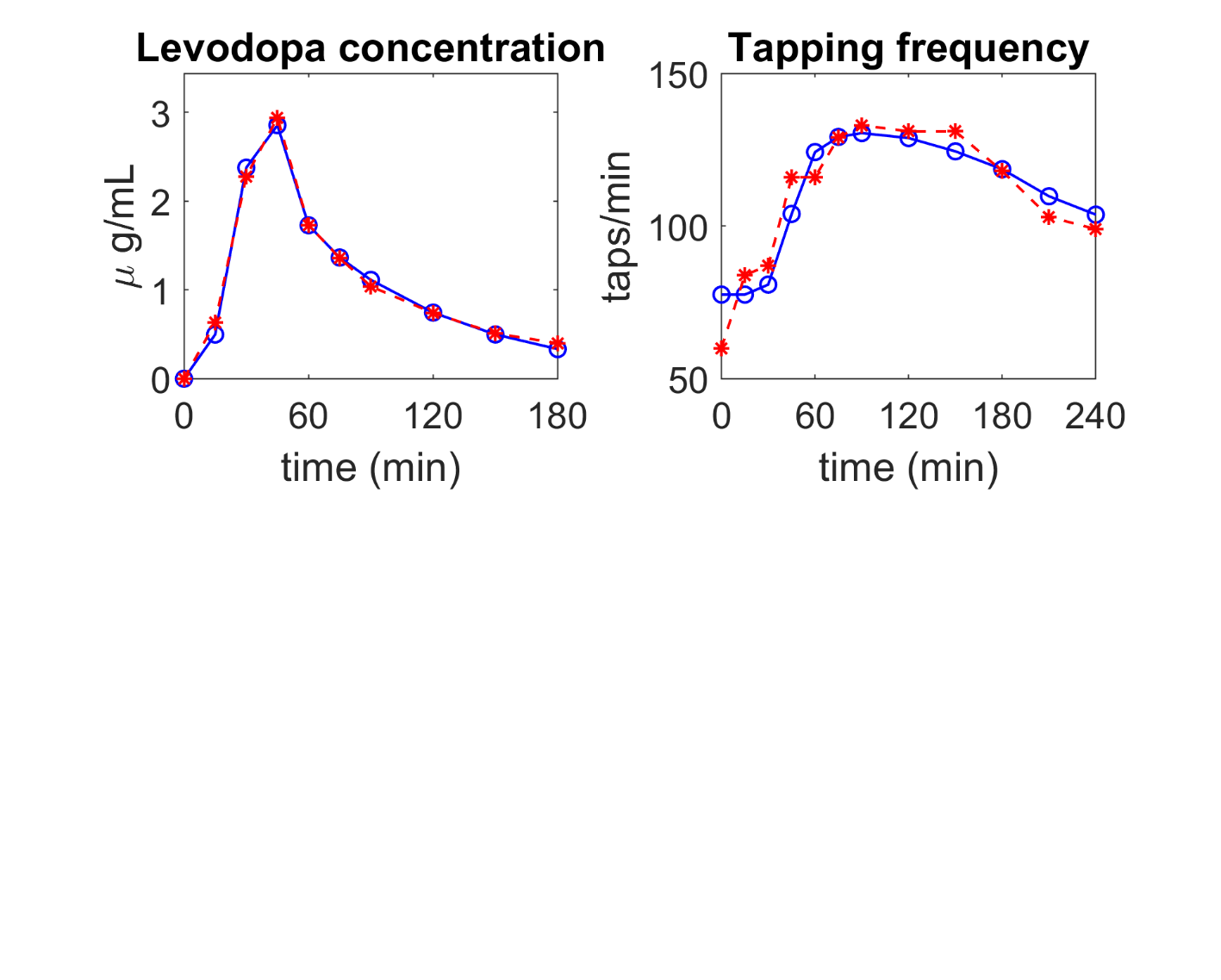


**Patient 4
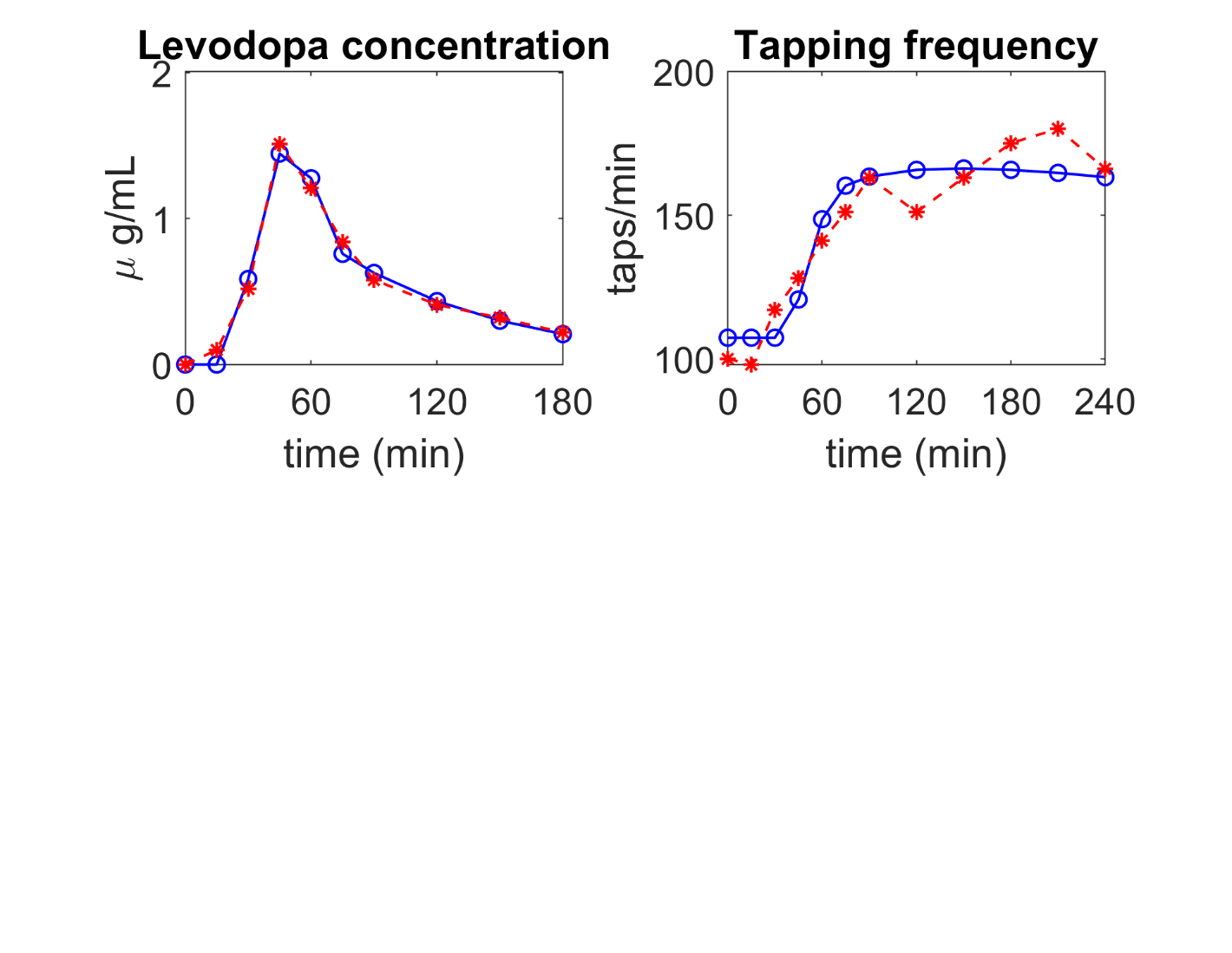
**

**Patient 5**

**
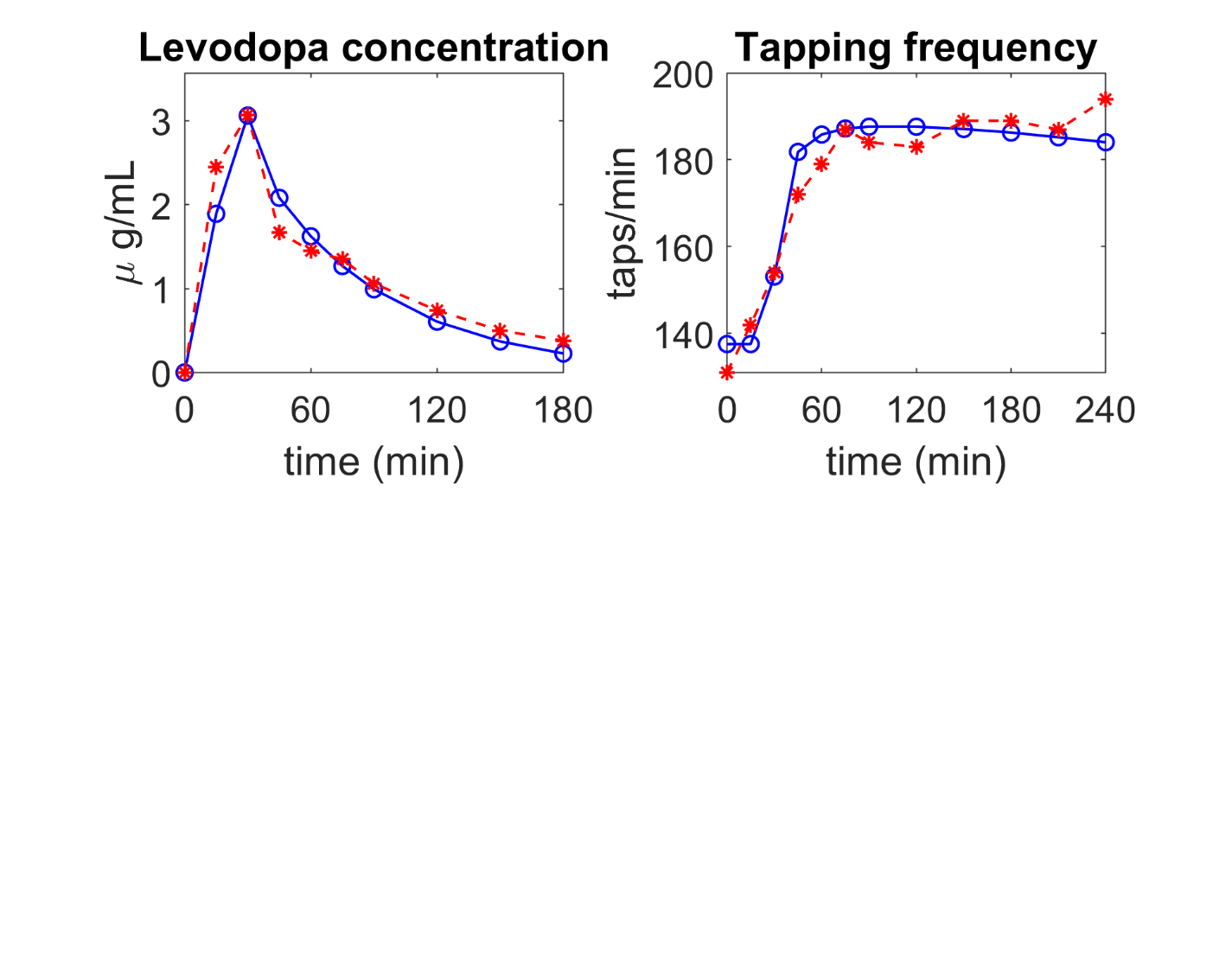
**

**Patient 6**

**
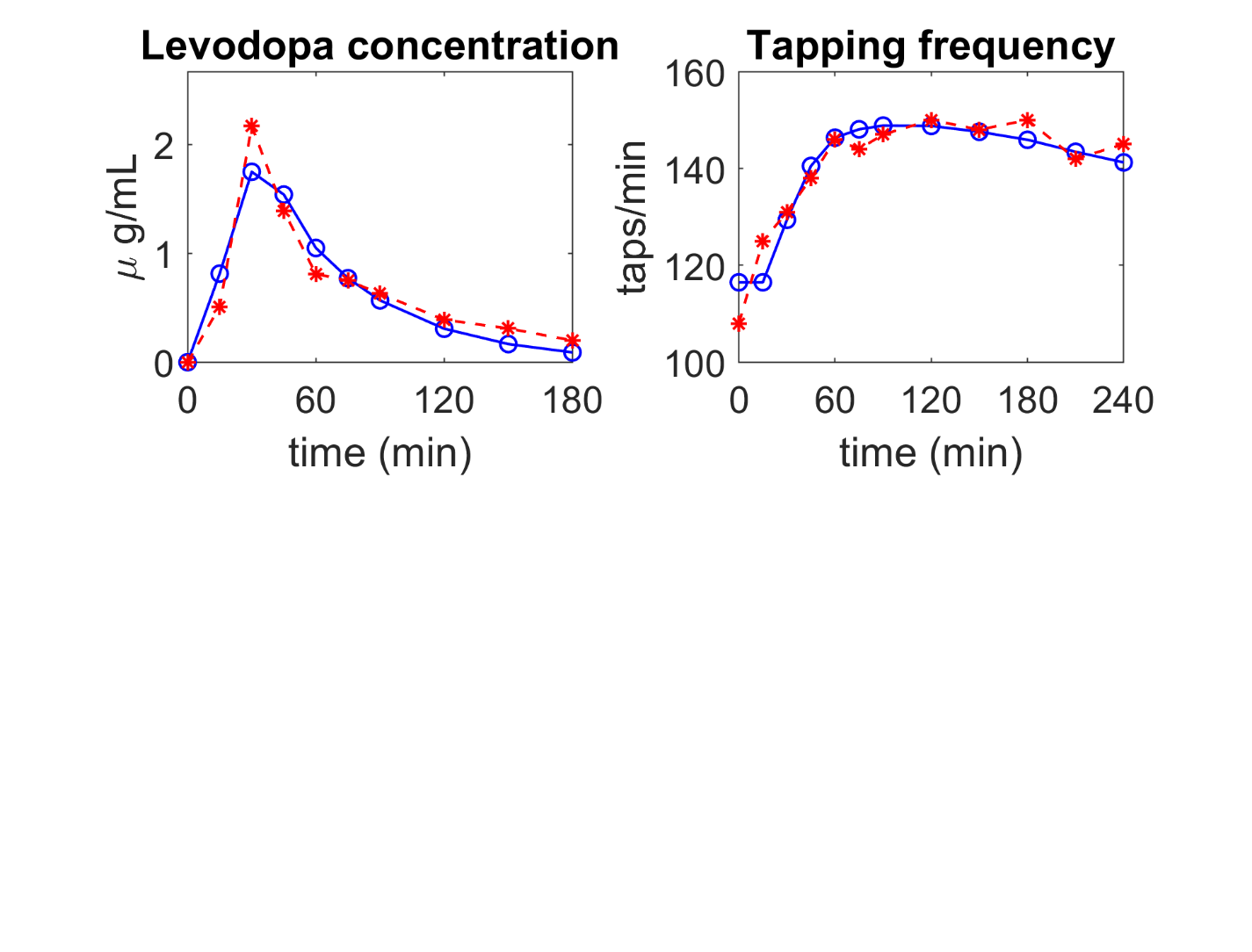
**

**Patient 7**

**
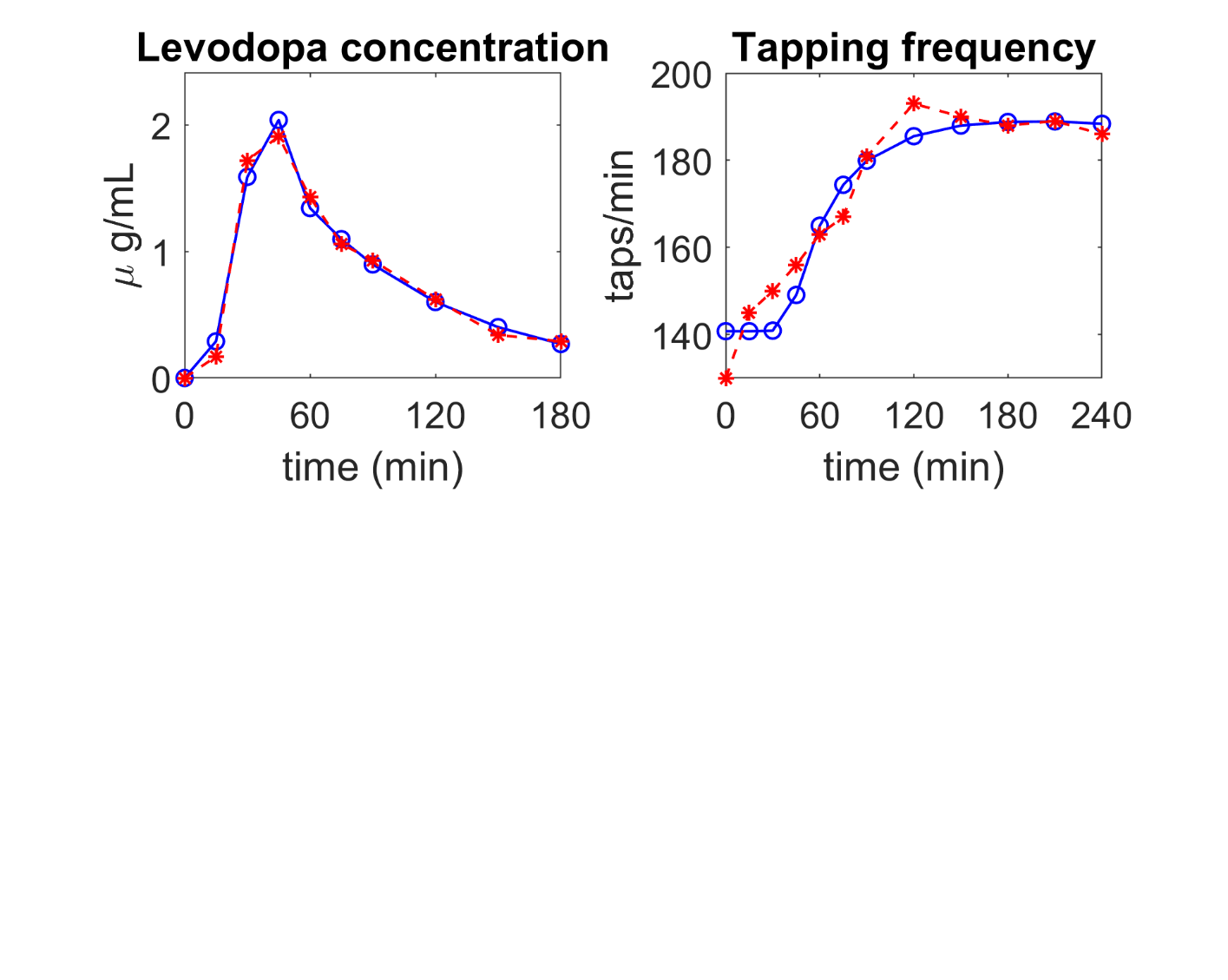
**

**Patient 8**

**
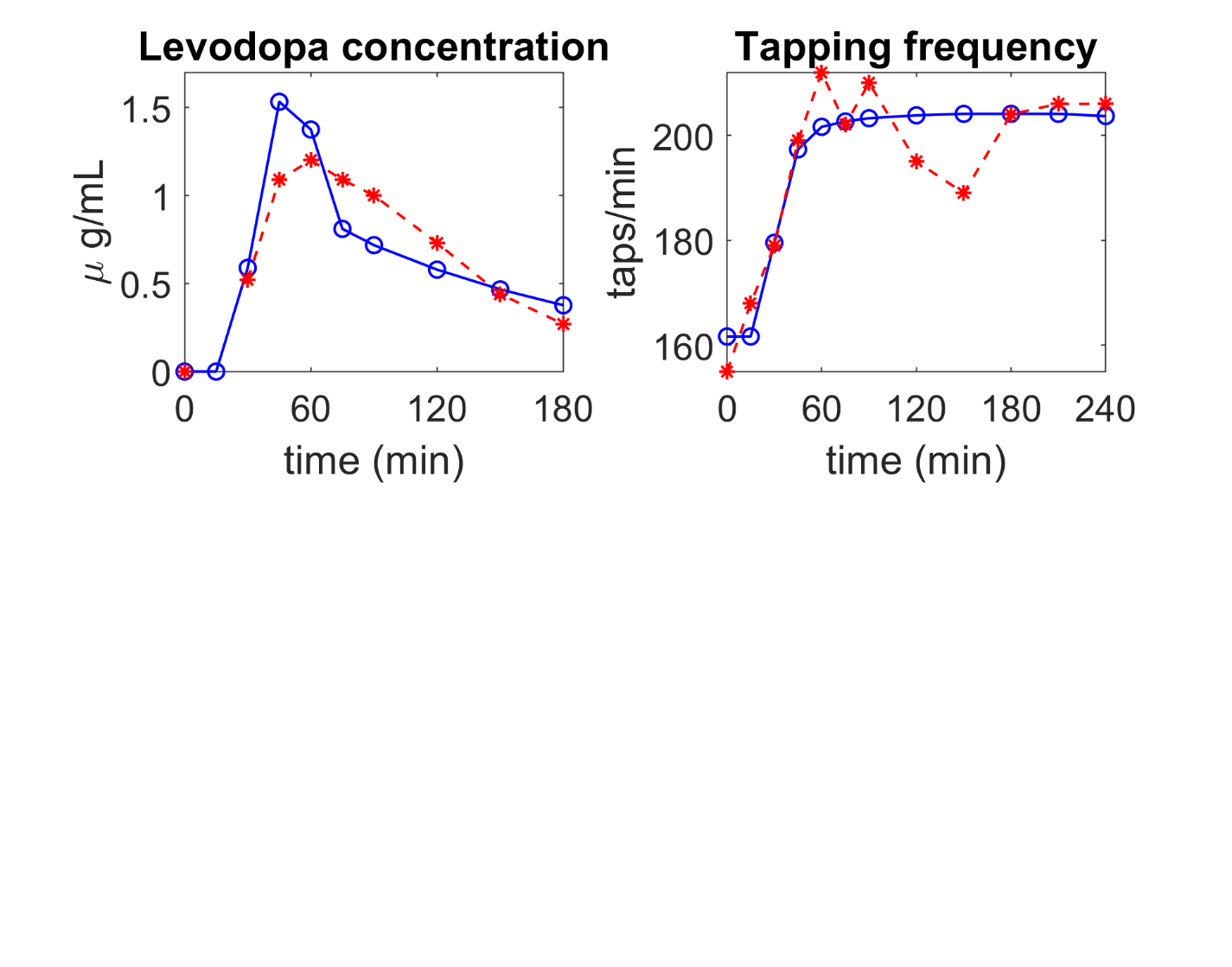
**

**Patient 9**

**
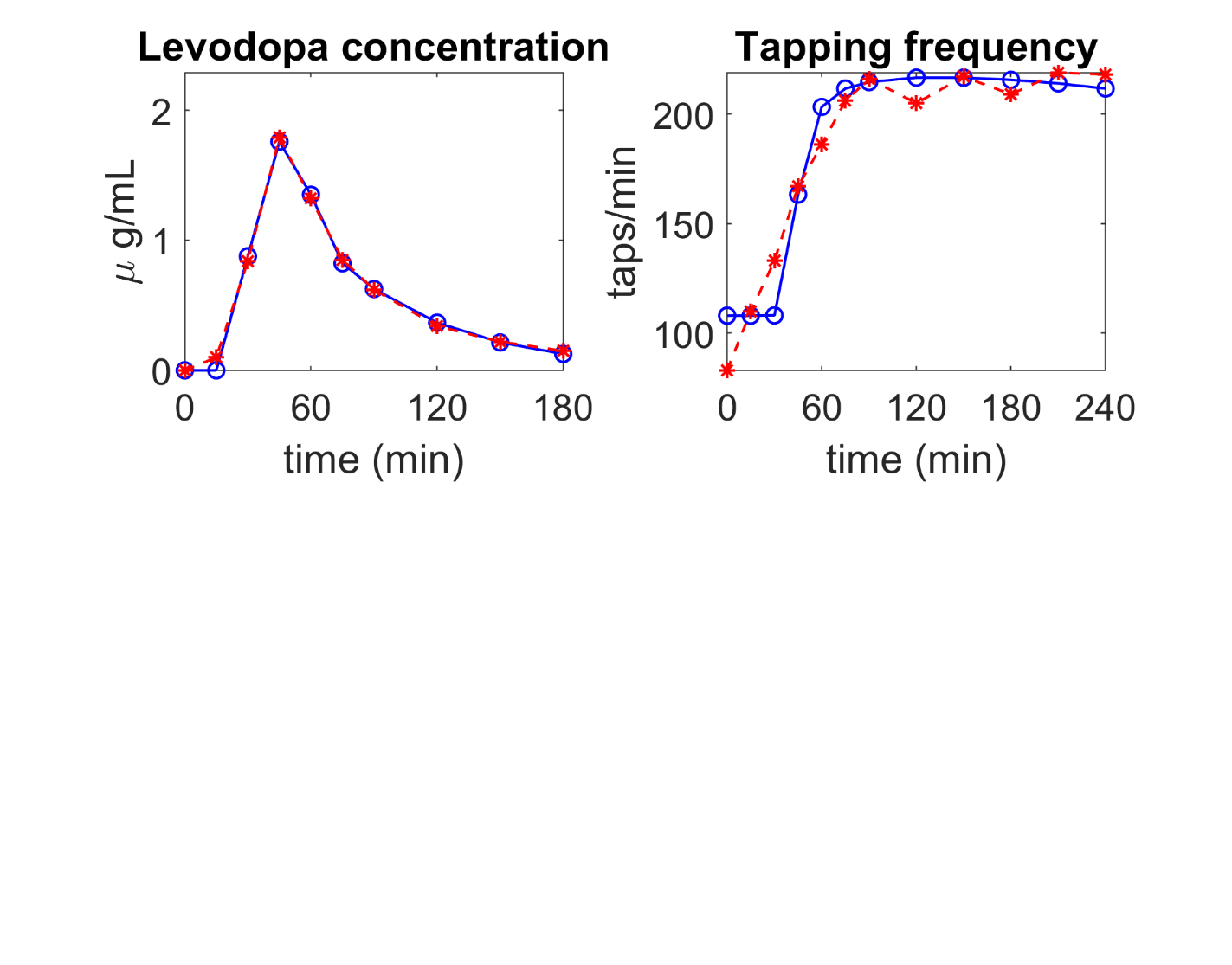
**

**Patient 10**

**
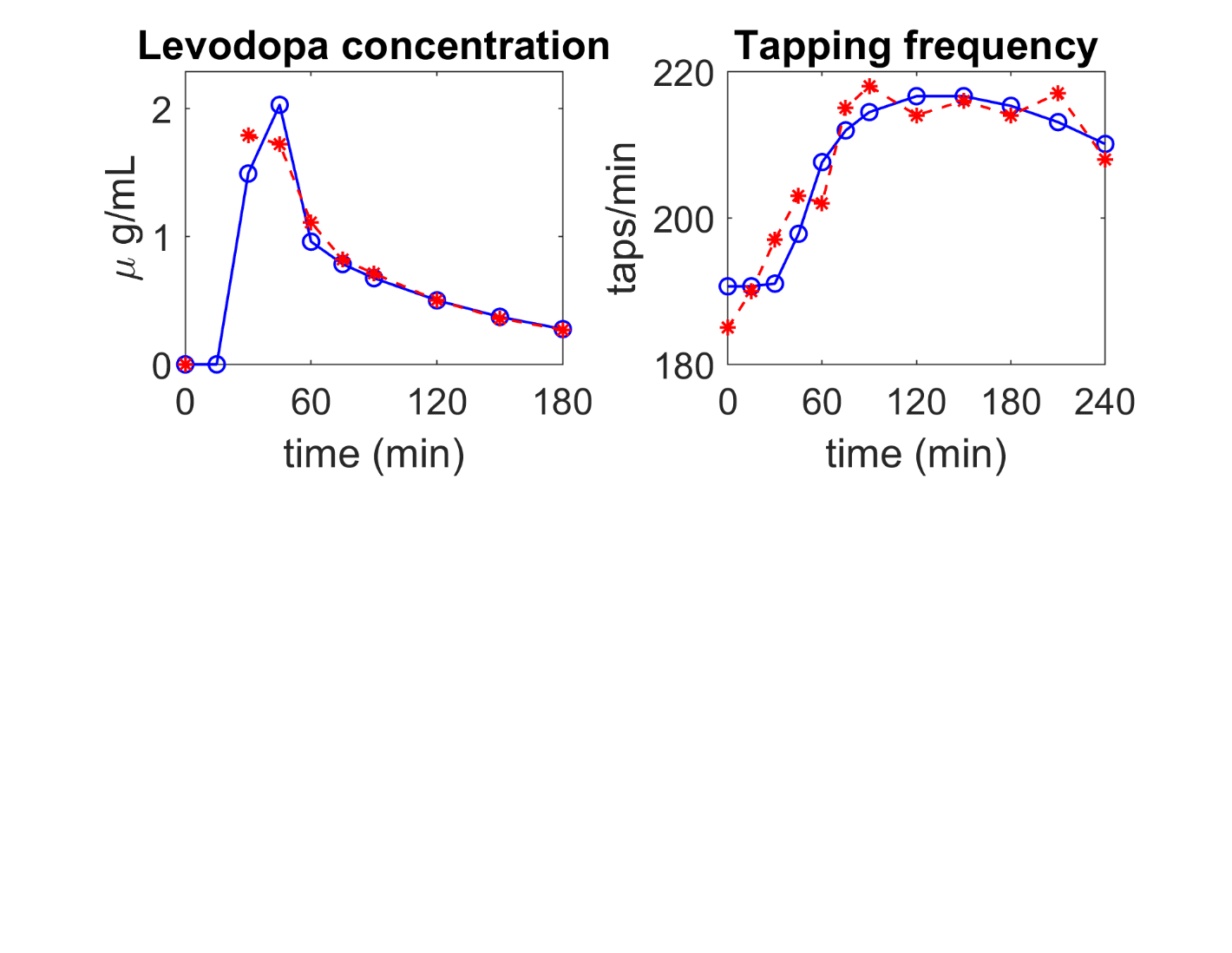
**

**Patient 11**

**
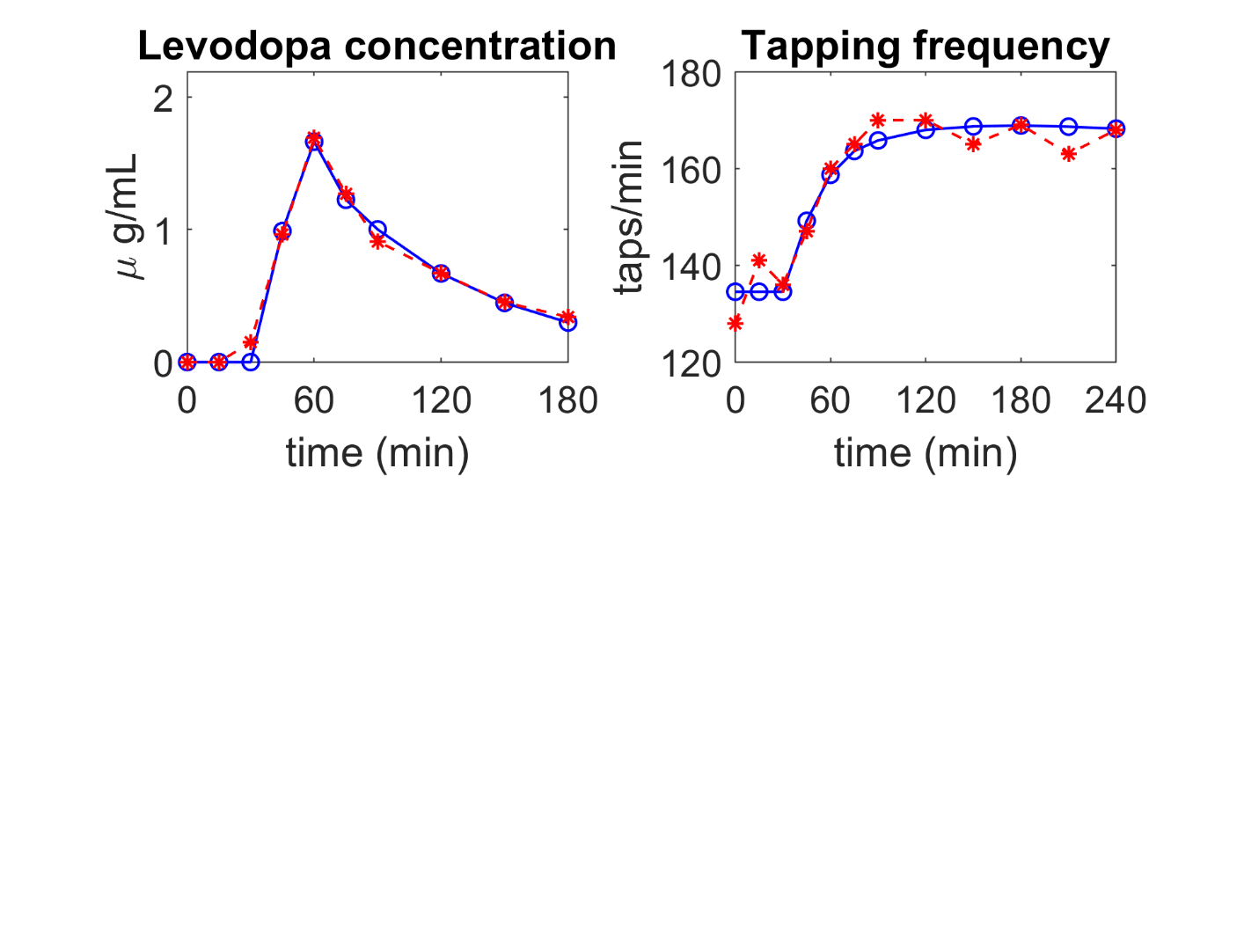
**

**Patient 12**

**
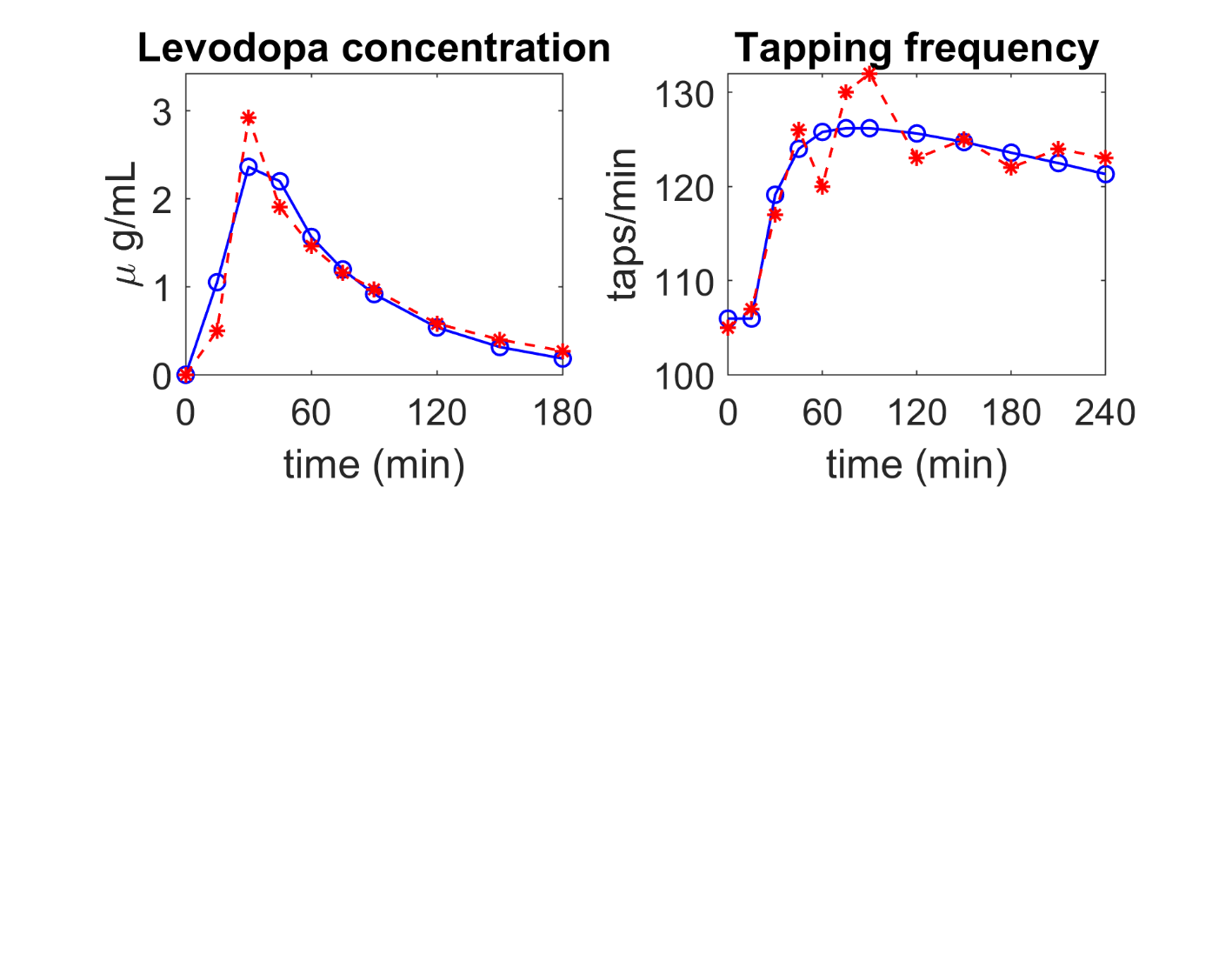
**

**Patient 13**

**
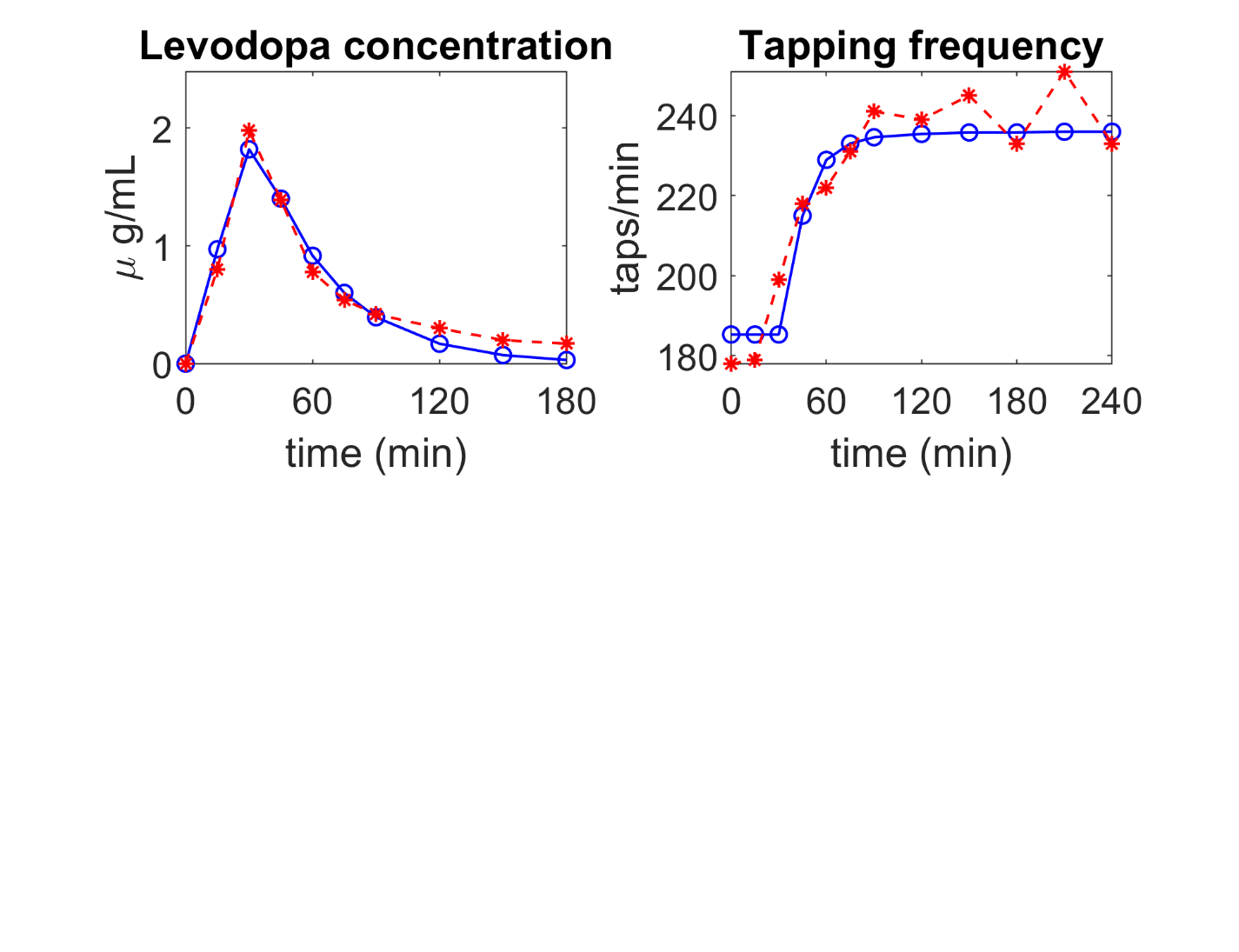
**

**Group 2 (fluctuating)**

**Patient 1**

**
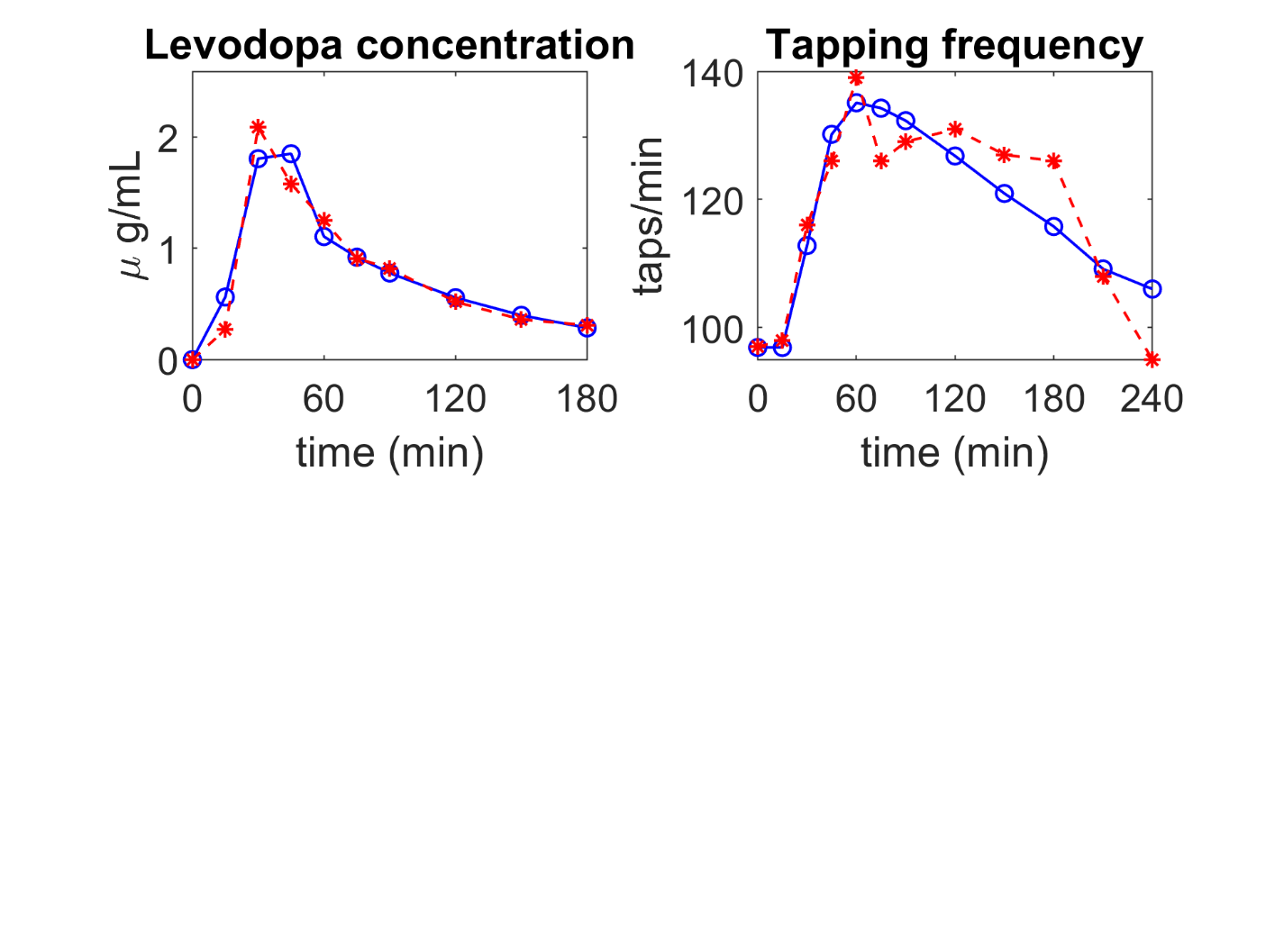
**

**Patient 2**

**
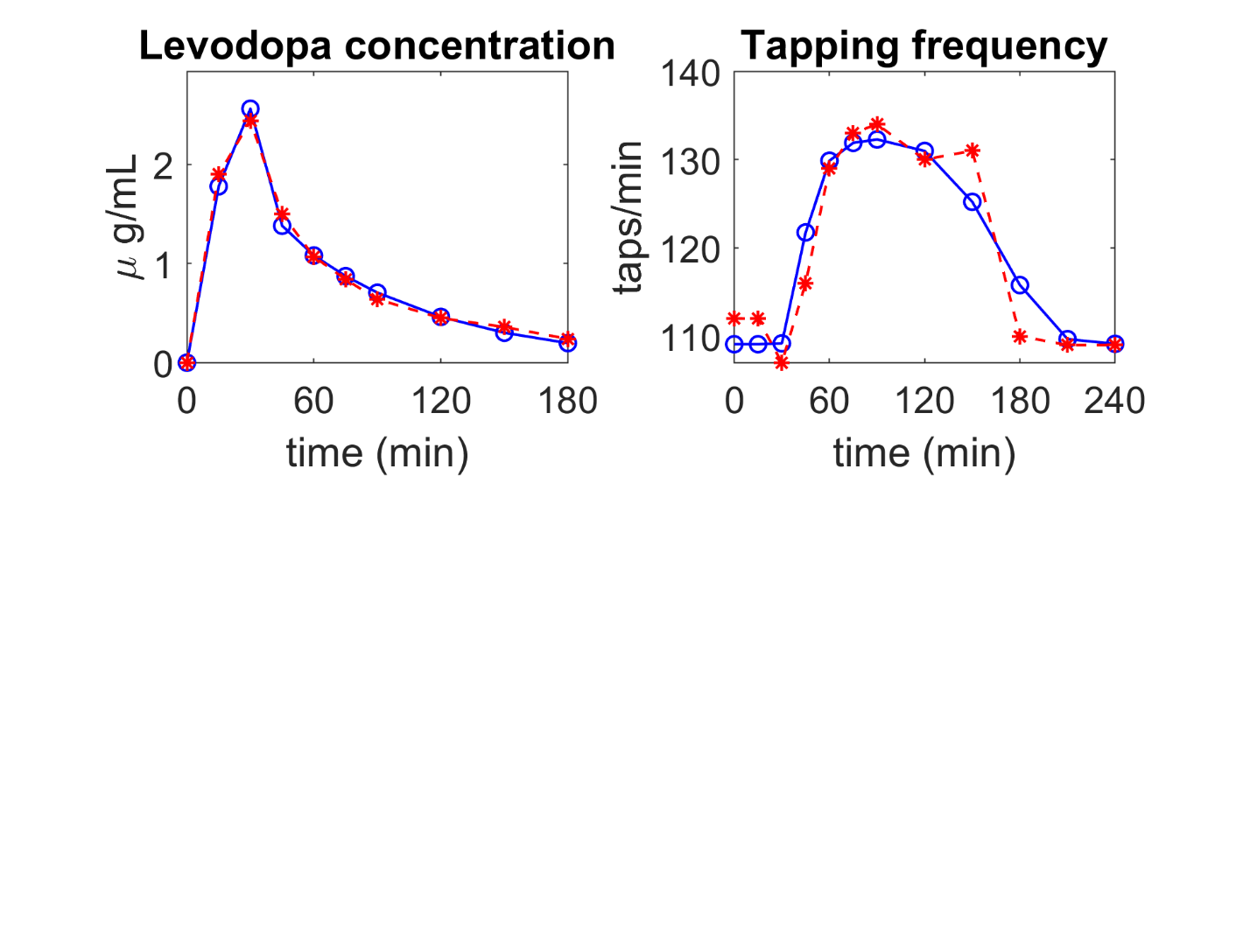
**

**Patient 3**

**
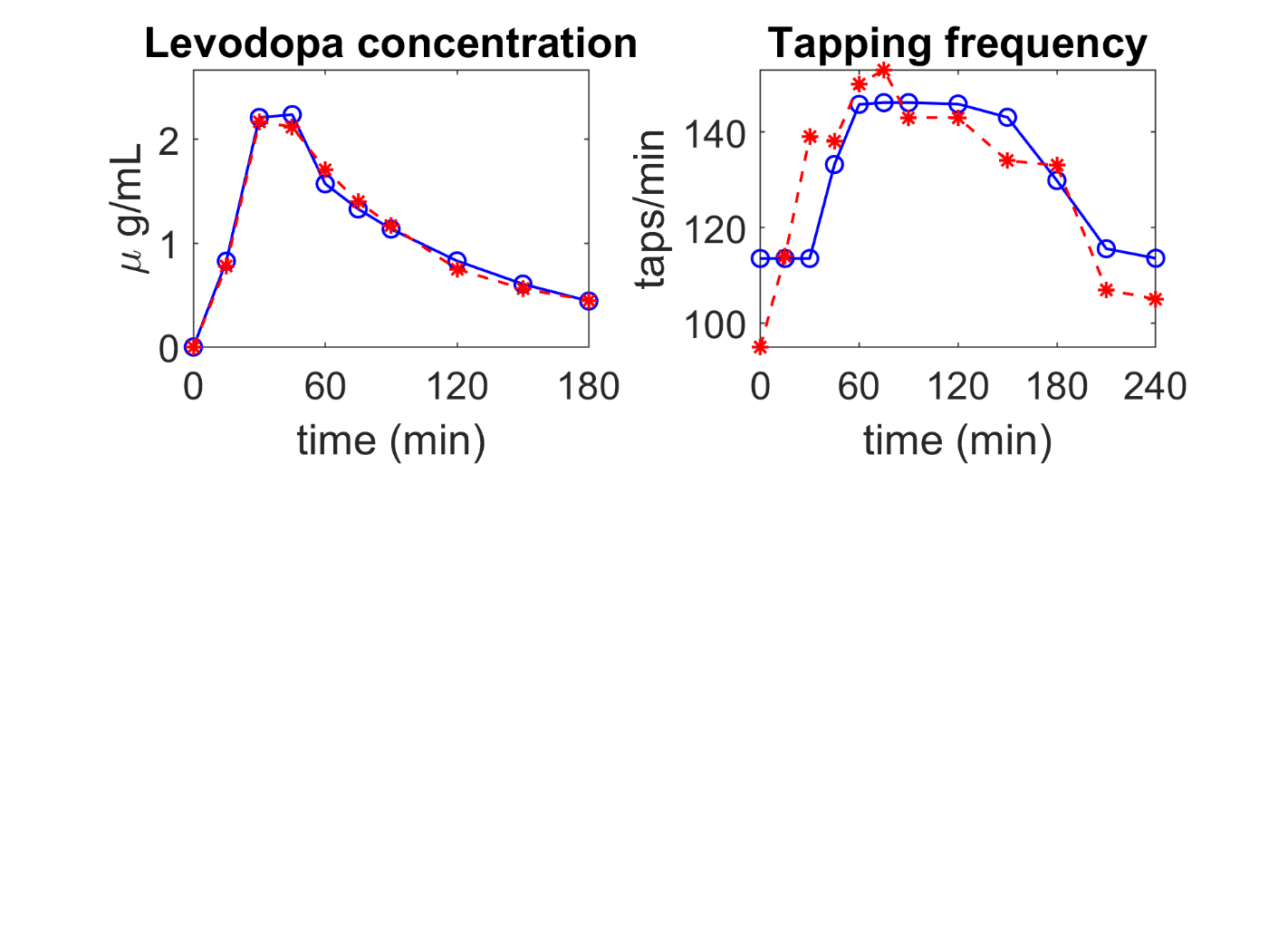
**

**Patient 4**

**
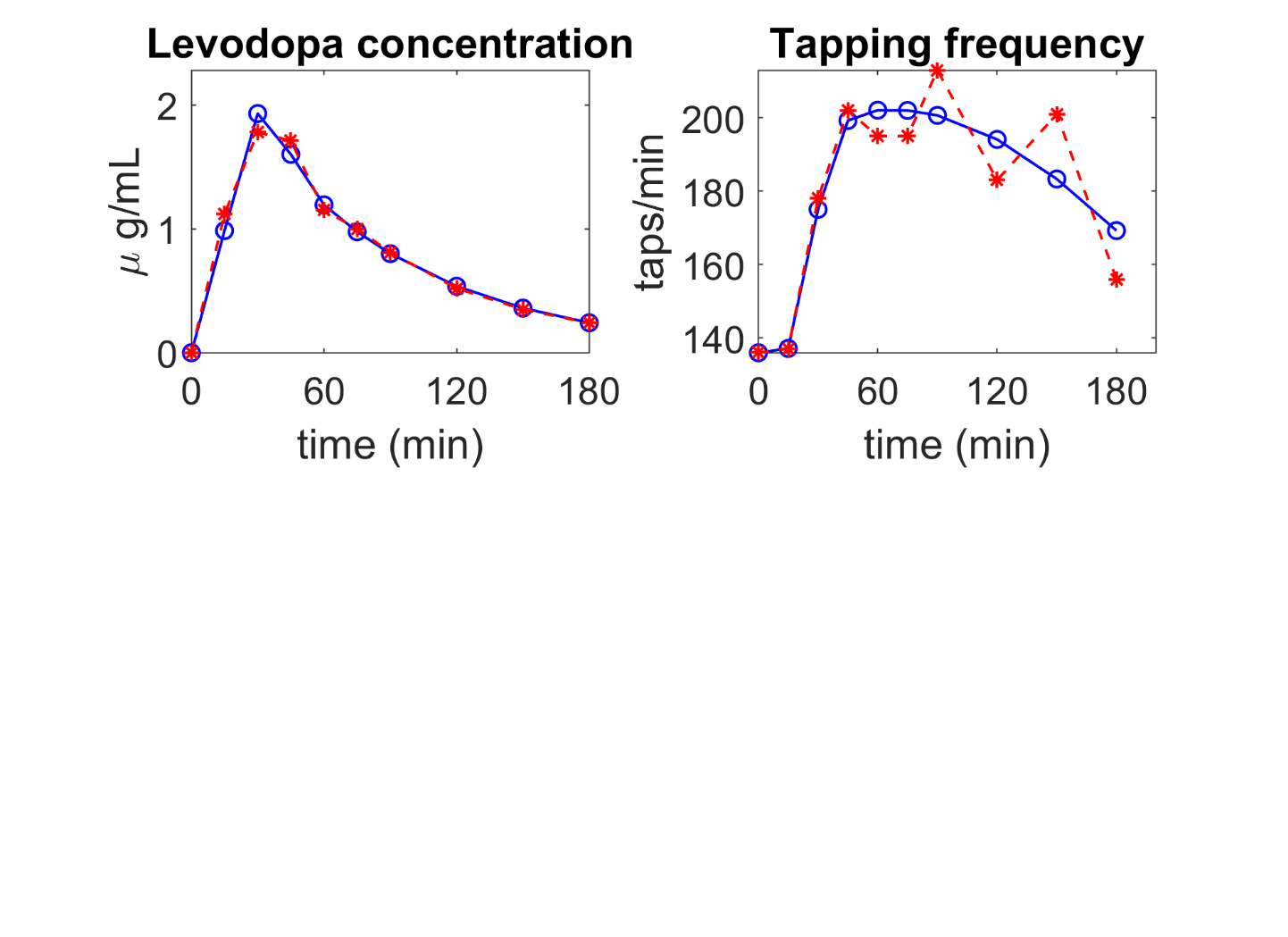
**

**Patient 5**

**
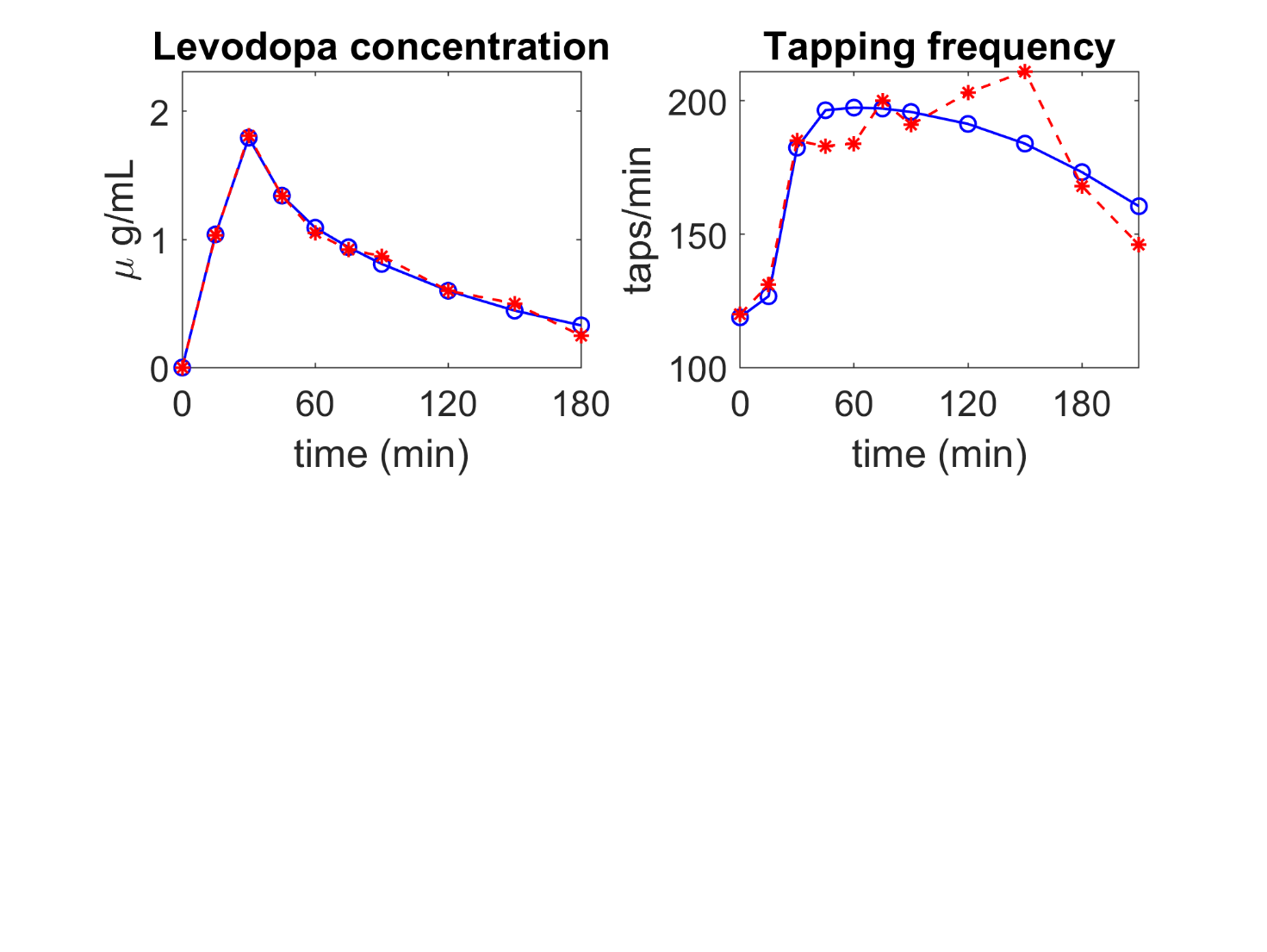
**

**Patient 6**

**
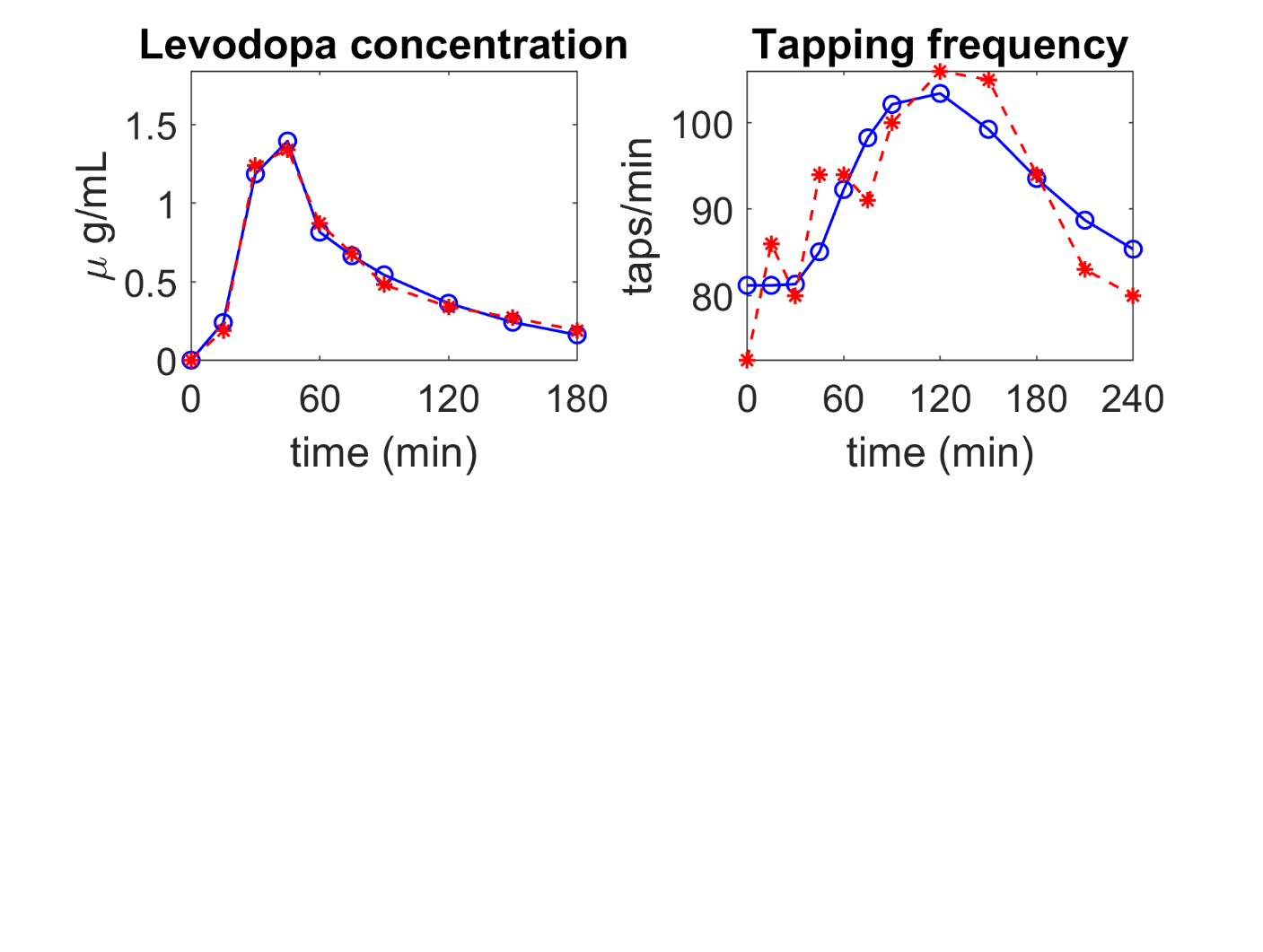
**

**Patient 7**

**
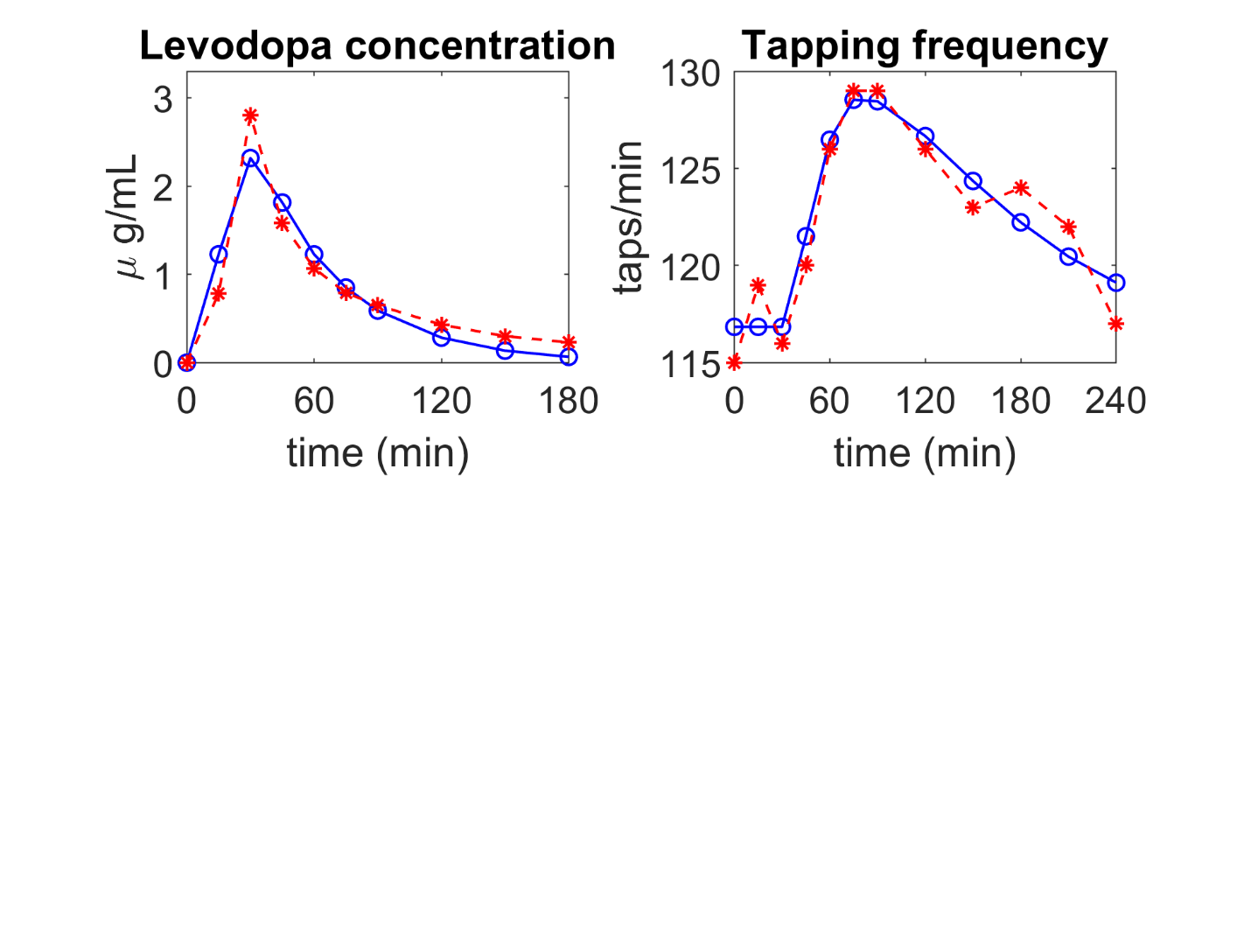
**

**Patient 8**

**
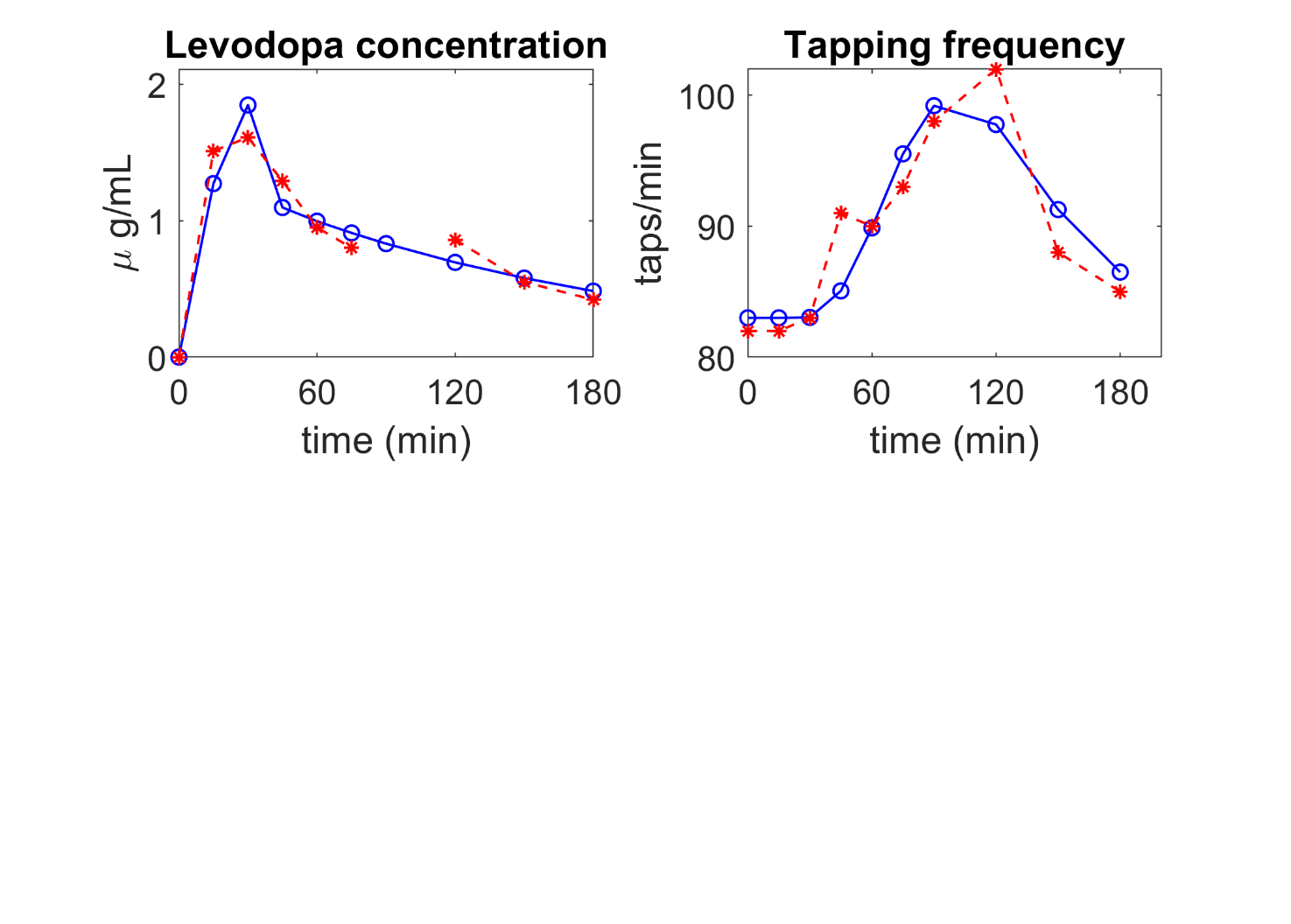
**

**Patient 9**

**
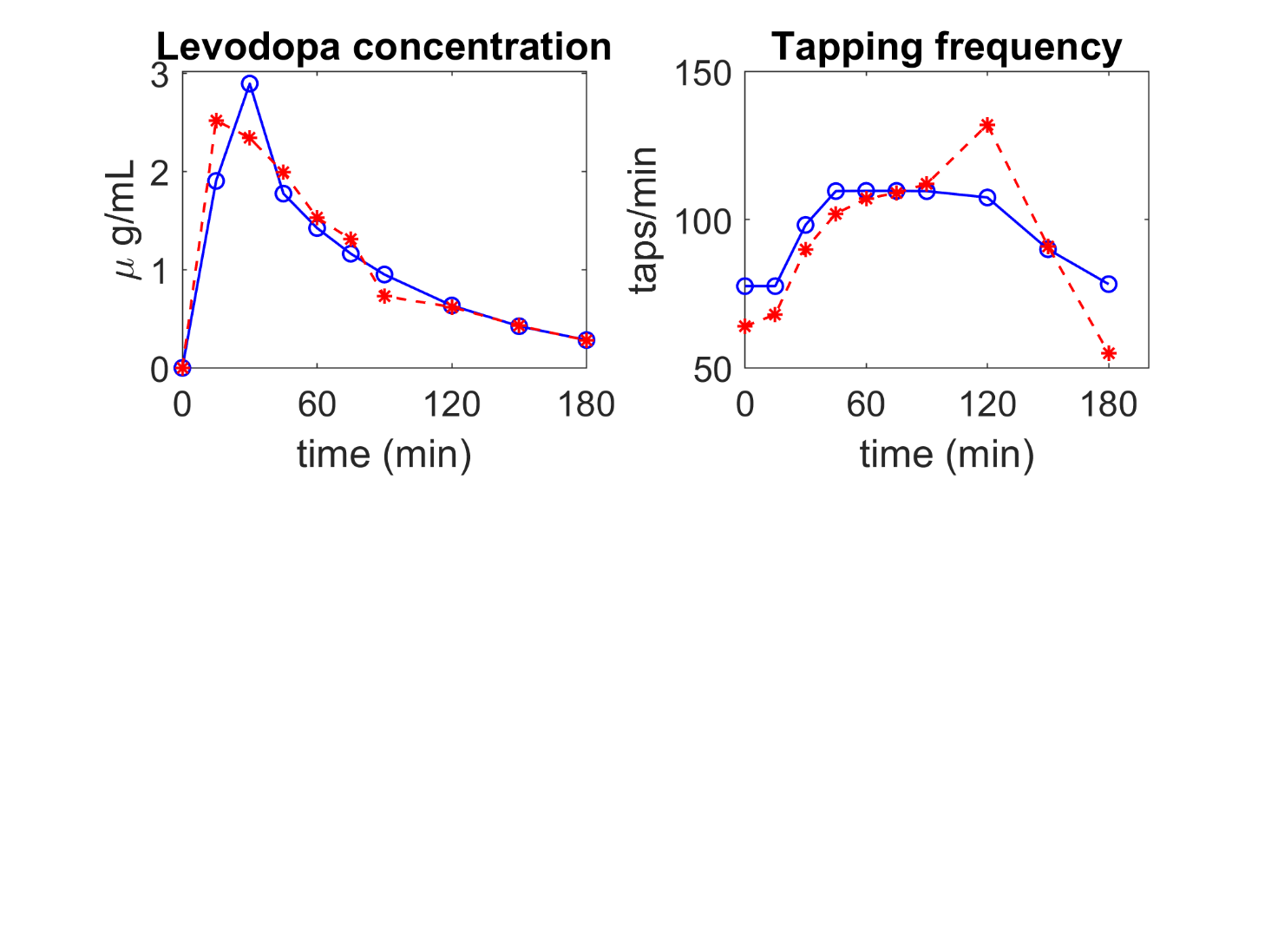
**

**Patient 10**

**
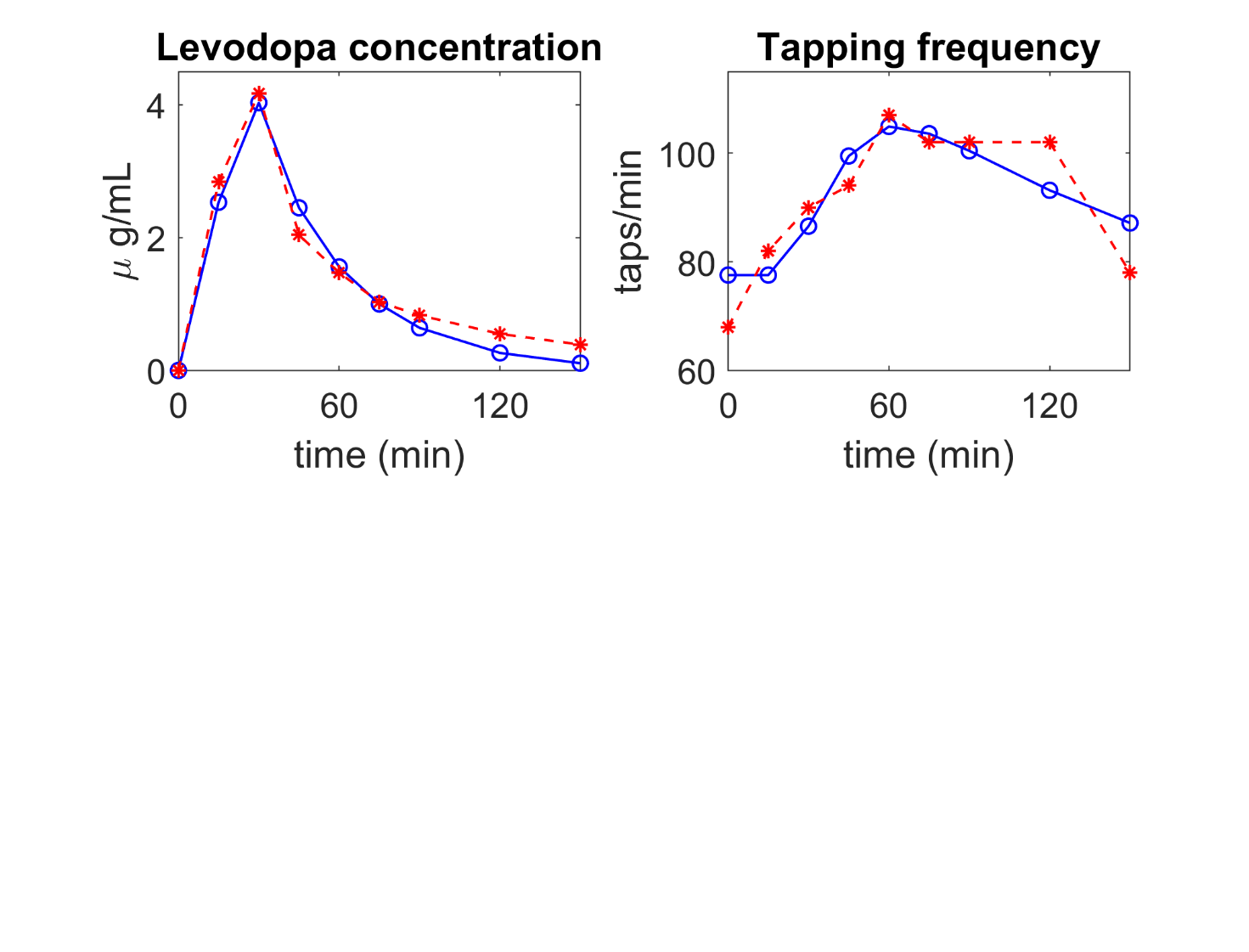
**

**Patient 11**

**
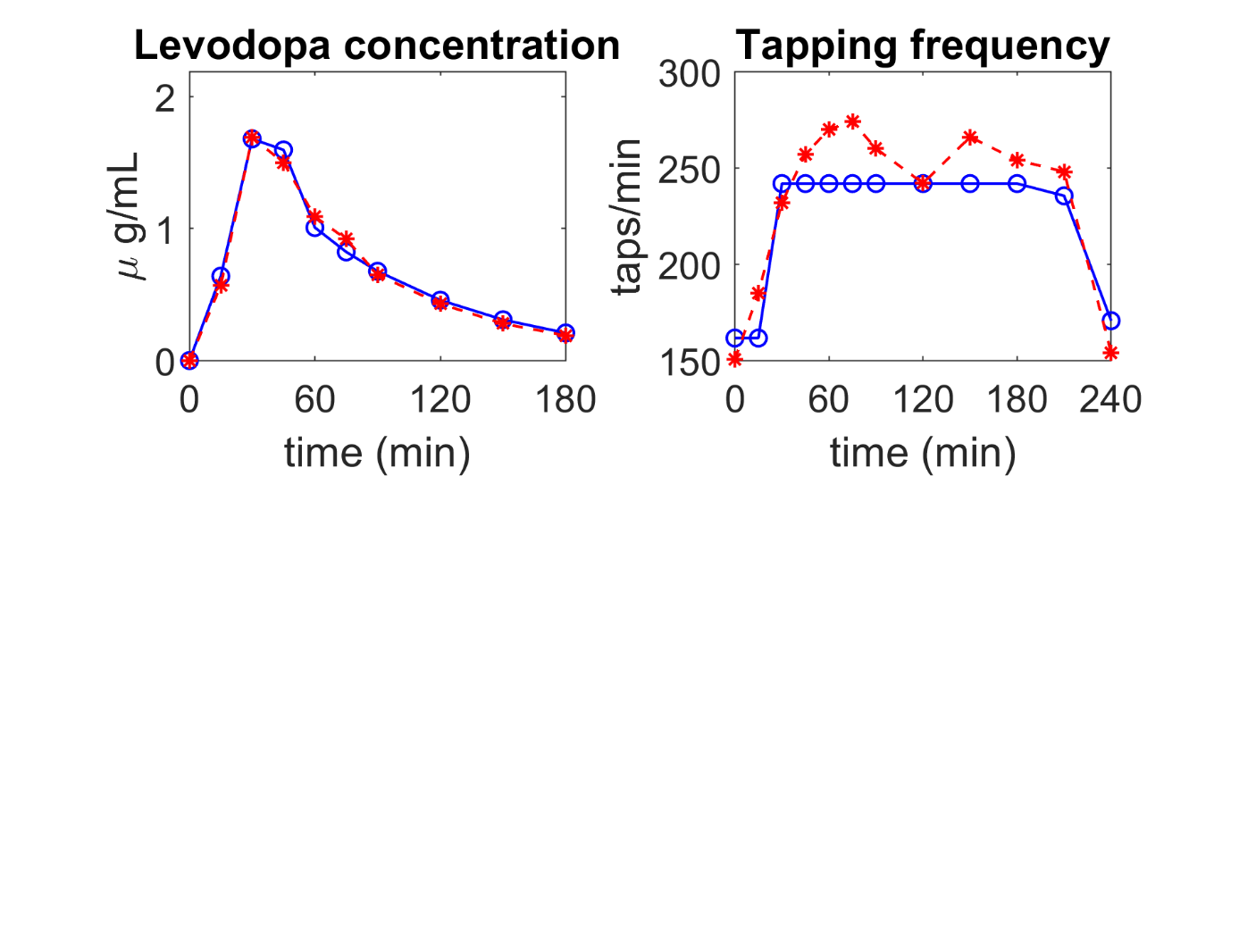
**

**Patient 12**

**
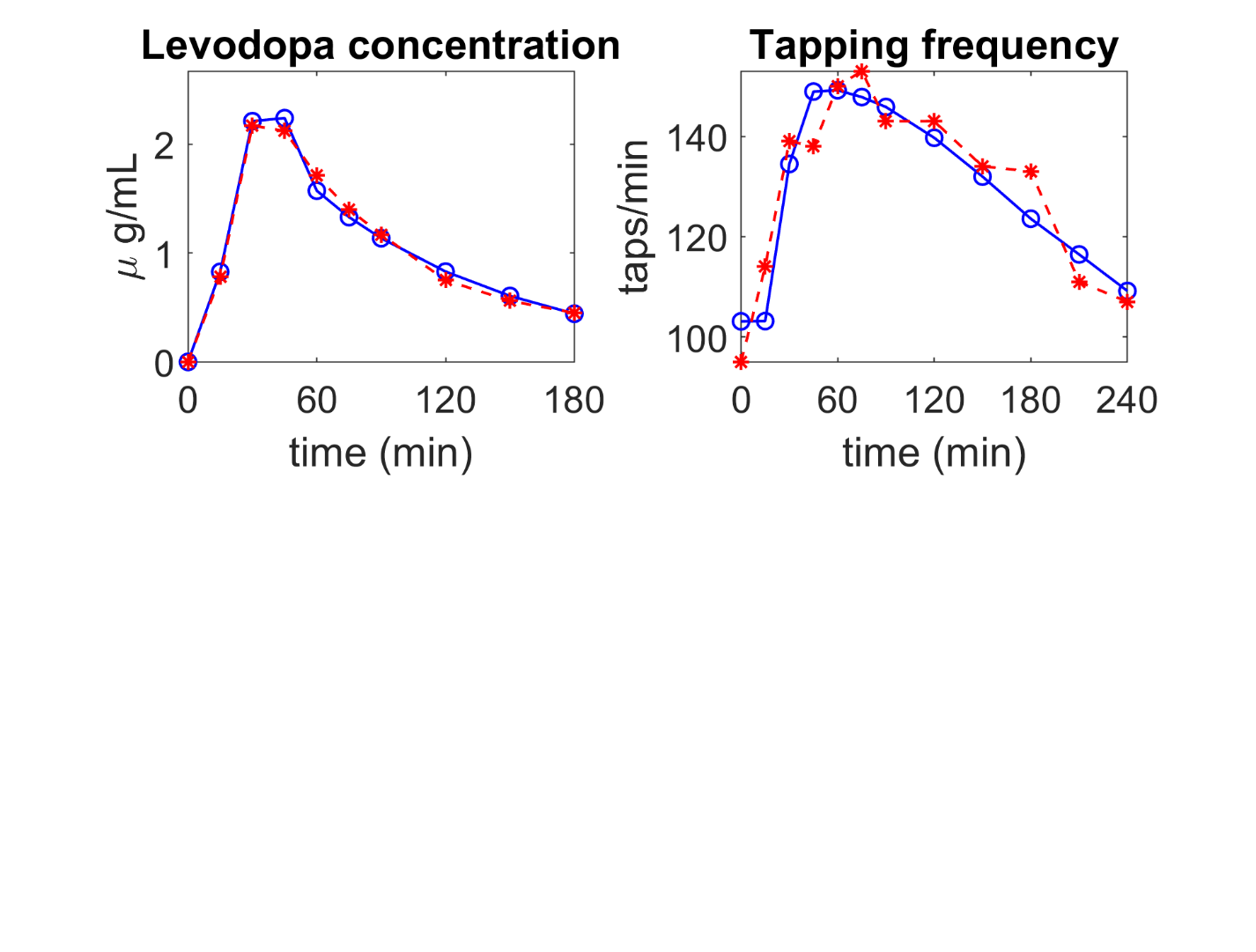
**

**Patient 13**

**
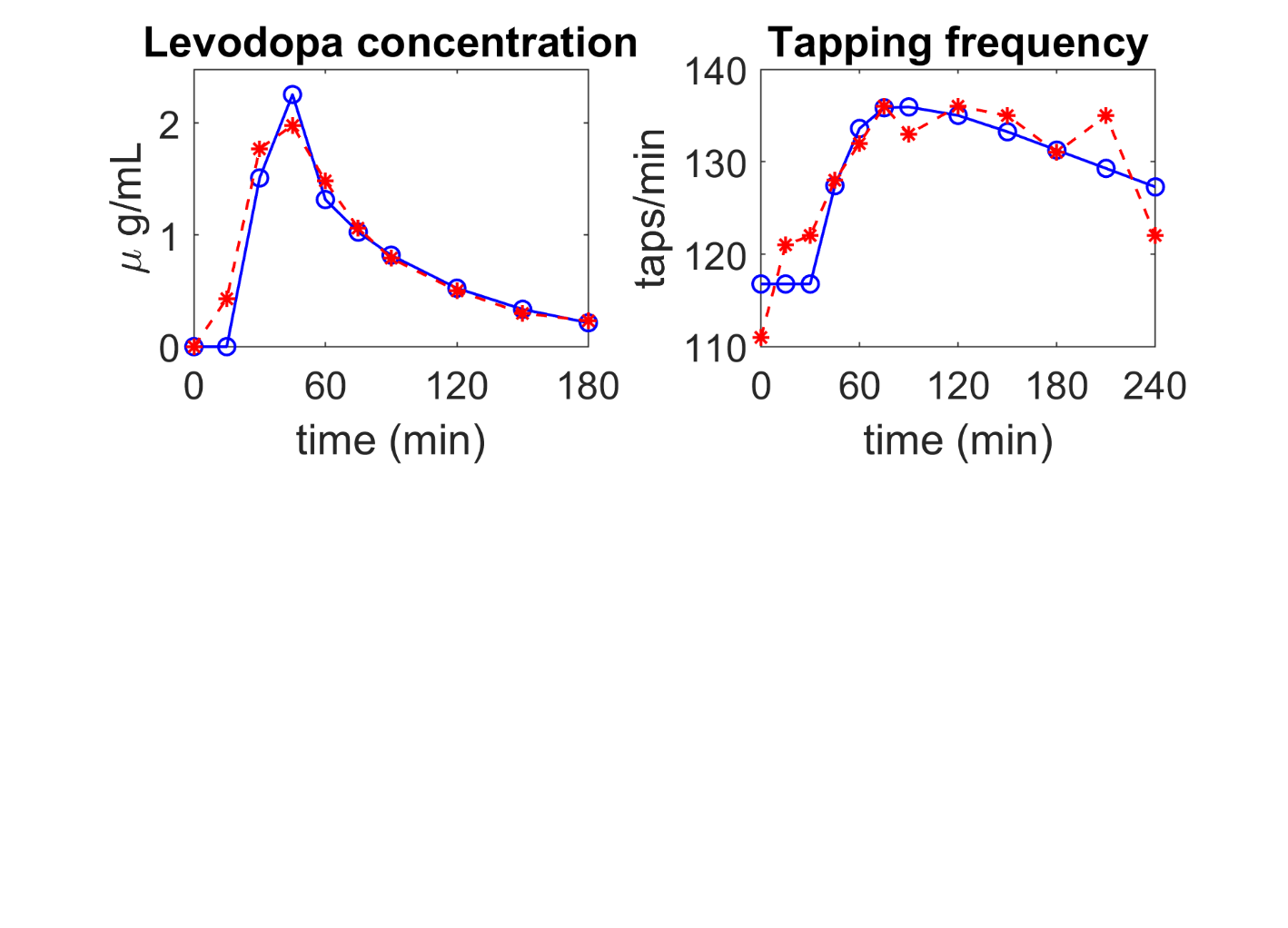
**
