# Supplementary material for: Mathematical modeling and parameter estimation of levodopa motor response in patients with parkinson disease
Source: PLoS One. 2020 Mar 3;15(3):e0229729. doi: 10.1371/journal.pone.0229729 (PMC7053720; doi:10.1371/journal.pone.0229729)
Supplement: S3 Material — (DOCX) [file pone.0229729.s003.docx]

**Supplementary Materials III: Sensitivity Analysis on Synapse changes**

**Mathematical modeling and parameter estimation of levodopa motor response in patients with parkinson disease**

**Mauro Ursino^1*^, Elisa Magosso^1^**^¶^**, Giovanna Lopane^2,3^**^¶^**, Giovanna Calandra-Buonaura^2,3^**^&^**, Pietro Cortelli^2,3^**^&^**, Manuela Contin^2,3^**^¶^

Simulations of two patients (patient1 of the stable group and patient2 of the fluctuating group) at different levels of the striatal synapses in the neurocomputational model. The pharmacodynamics parameters have been maintained at the values previously estimated on that patient. The synapses have been obtained using the training procedure illustrated in Ursino and Baston, Eur. J. Neurosci. 2018 at different epochs of training, starting from an initially untrained network (see Fig. 3 in that paper). Since a change in synapses significantly modifies the basal value of tapping frequency (a longer training is reflected in a higher tapping rate) the value of parameter D_0_ has been recalculated in each simulation, to maintain an initial tapping frequency as closely as possible to the basal frequency shown by the patient. In these conditions, the temporal pattern of tapping frequency is not significantly affected by the synapse level. In particular, differences between the two groups cannot be explained by synapse alterations.

**Patient 1 (stable group) Patient 2 (fluctuating group)**

**
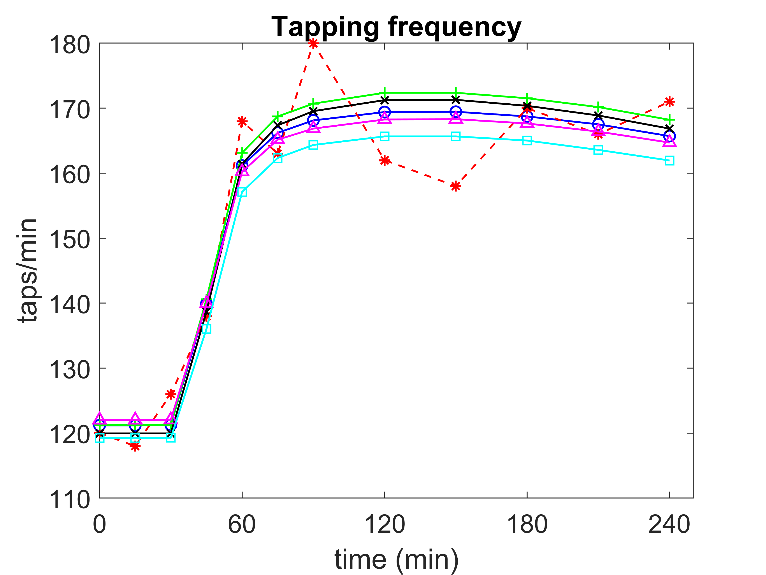
**
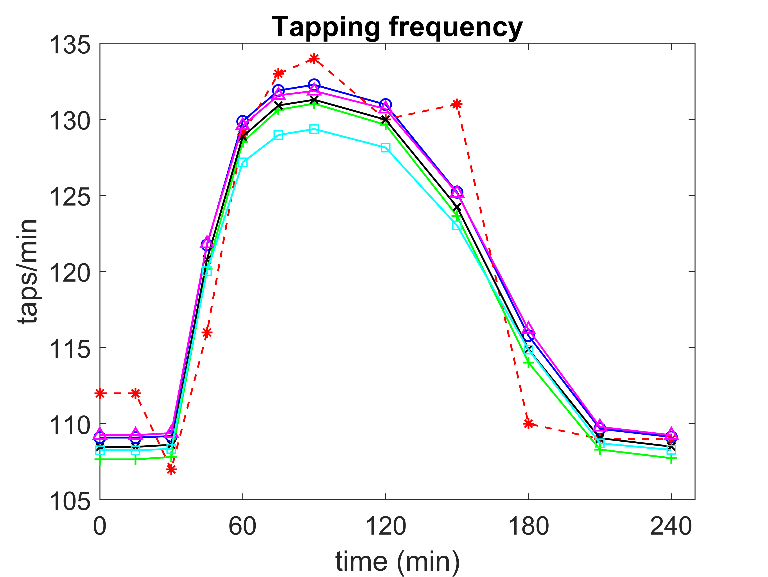


Dashed red lines with asterisks: patient data. Continuous lines with symbols: model simulation results after 60 (cyanus 🞎), 80 (magenta Δ) 100 (blue o), 150 (green +) and 200 (black x) epochs of training.
